# Accelerated cartilage regeneration via chondrocyte metabolic reprogramming using nano-steroid-conjugated mesenchymal stem cells in osteoarthritis

**DOI:** 10.7150/thno.120765

**Published:** 2026-01-01

**Authors:** Jong Yeong Lee, Jun Young Park, In Ah Kwon, Su Hyun Lim, Ki Bum Kim, Jun-Young Park, Youn Joo Kang, Sang-Hyun Kim, Dongwoo Khang, Jin Kyeong Choi

**Affiliations:** 1 Department of Immunology, Jeonbuk National University Medical School, Jeonju 54907, South Korea.; 2 Lee Gil Ya Cancer and Diabetes Institute, Gachon University, Incheon 21999, South Korea.; 3 Department of Health Sciences and Technology, GAIHST, Gachon University, Incheon 21999, South Korea.; 4 Ectosome Inc., Incheon 21984, South Korea.; 5 Department of Orthopaedics Surgery, Jeonbuk National University Medical School, Jeonju 54907, South Korea.; 6 Department of Biochemistry, Chungbuk National University, Cheongju, 28644, South Korea.; 7 Department of Rehabilitation Medicine, Nowon Eulji Medical Center, Eulji University, Seoul 01830, South Korea.; 8 CMRI, Department of Pharmacology, School of Medicine, Kyungpook National University, Daegu 41944, South Korea.; 9 Department of Physiology, College of Medicine, Gachon University, Incheon 21999, Republic of Korea.; 10 Biomedical Research Institute of Jeonbuk National University Hospital, Institute for Medical Sciences, Jeonbuk National University, Jeonju 54907, South Korea.

**Keywords:** mesenchymal stem cells, gold nanostars, osteoarthritis, chondrocytes, metabolic reprogramming

## Abstract

**Background:** Osteoarthritis (OA) is a prevalent and debilitating chronic disease for which there are currently no approved disease-modifying osteoarthritis drugs (DMOADs). While mesenchymal stem cells (MSCs) have emerged as promising DMOAD candidates, their clinical application is hindered by inconsistent *in vivo* efficacy and an incomplete understanding of the underlying pathological mechanisms and therapeutic targets.

**Methods:** To address these limitations, we developed a novel therapeutic strategy by conjugating MSCs with steroid-loaded gold nanostars (MSC-Au-Steroid). The effects of MSC-Au-Steroid were assessed *in vitro* using osteoarthritis patient-derived chondrocytes and peripheral blood mononuclear cells (PBMCs), and *in vivo* using a monosodium iodoacetate (MIA)-induced mouse model of osteoarthritis. Key assessments included anti-inflammatory activity, metabolic profiling (glycolysis and oxidative phosphorylation), mitochondrial function, reactive oxygen species (ROS) production, mTOR signaling, and immunomodulatory effects on Th1, Th17, and regulatory T cells (Tregs).

**Results:** MSC-Au-Steroid demonstrated strong anti-inflammatory effects in OA chondrocytes, promoted cartilage regeneration, and normalized altered metabolic profiles under inflammatory conditions. It improved mitochondrial function and suppressed excessive ROS production via mTOR signaling regulation. *In vivo*, MSC-Au-Steroid alleviated clinical symptoms and preserved cartilage integrity in the MIA-induced OA mouse model. Furthermore, it exhibited immunomodulatory effects in both mouse and patient-derived PBMCs, inhibiting Th1 and Th17 responses while promoting Treg induction and restoring immune tolerance.

**Conclusions:** MSC-Au-Steroid represents a promising multifactorial therapeutic candidate for OA by targeting both metabolic reprogramming and immune modulation. These findings provide strong evidence that MSC-Au-Steroid acts as a disease-modifying agent, addressing multiple pathological factors and offering superior efficacy compared to current therapies.

## Introduction

Osteoarthritis (OA), a leading degenerative joint disease affecting hundreds of millions of people worldwide, is characterized by cartilage damage, joint deformities, and chronic pain 1, 2. The pathological features of OA include progressive loss of articular cartilage, changes in subchondral bone, synovial inflammation, and development of joint deformities 1, 2. The progression of this disease significantly affects daily activities and quality of life, and its prevalence continues to increase in aging societies 3. Current treatments for OA primarily focus on pain relief and functional improvement; however, no treatment has been successful in halting disease progression or cartilage regeneration 4. Commonly used medications such as non-steroidal anti-inflammatory drugs (NSAIDs), opioid analgesics, steroids, biologics, and intra-articular injections such as hyaluronic acid only provide temporary relief and limited long-term efficacy with potential side effects 4, 5. Surgical methods such as joint replacement are effective for severe cases but are associated with risks of post-surgical complications and high costs 5. Therefore, novel disease-modifying osteoarthritis drugs (DMOADs) are urgently needed.

Mesenchymal stem cells (MSC) have potential as DMOADs because of their ability to self-renew and differentiate into chondrocytes, as well as their capacity to target immune cells, such as CD4^+^ T cells, B cells, NK cells, and monocytes, to alleviate joint inflammation, and maintain peripheral tolerance 6, 7. MSC injection therapy has the advantage of being less invasive and offering shorter recovery periods compared to those of surgical procedures 8. However, the biological mechanisms of MSC in OA remain unclear and evidence regarding their long-term efficacy remains insufficient 9. Moreover, the inflammatory environment in OA may impair MSC survival and function, potentially reducing their therapeutic effectiveness. Additionally, MSC have limitations in precisely reaching and distributing to damaged cartilage areas 8, 10, 11. To overcome these challenges, appropriate cell support strategies are required to enhance the efficacy of MSC.

Given that OA progresses owing to inflammatory cytokines and mediators that cause joint tissue damage, leading to mechanical failure of the joint and disease progression, identifying and targeting these pathways at an early stage is critical 3. Accordingly, we leveraged recent advances in the biomedical applications of nanoparticles to introduce an innovative therapeutic strategy for controlling severe joint inflammation by enabling MSC to precisely target inflammation and effectively deliver anti-inflammatory drugs without genetic modifications. Specifically, a model of MSC “educated” to be effectively recruited to inflammation sites (MSC), conjugated the anti-inflammatory drug triamcinolone (steroid) with star-shaped gold nanoparticles (Au) to create Au-Steroid, and then attached this to MSC using anti-CD90 antibodies bound to the CD90 membrane protein on the MSC surface, generating MSC-Au-Steroid 12. According to our previous report, MSC-Au-Steroid demonstrated enhanced drug delivery capacity, strengthened anti-inflammatory effects, and improved pain suppression compared with those of MSC or steroid (TA) in rheumatoid arthritis (RA) animal models 12. However, despite the clear anti-inflammatory effects of MSC-Au-Steroid on arthritis, the mechanisms underlying accelerated cartilage regeneration remain unclear. In this regard, metabolic restoration needs to be simultaneously considered for both chondrocytes and immune cells due to their critical roles in the OA model. In OA, chondrocytes and immune cells undergo metabolic shifts to compensate for impaired metabolism, providing the energy required for inflammatory responses and enhancing interactions with the immune system by promoting a shift from oxidative phosphorylation to glycolysis 13. During this process, mitochondria generate excessive reactive oxygen species (ROS) from oxidative phosphorylation, leading to mitochondrial dysfunction, activation of inflammatory pathways, and, consequently, chondrocyte degeneration 5, 14. In parallel, the mTOR signaling pathway, a key regulator of cellular metabolism, is significantly upregulated in chondrocytes from patients with OA and in mouse models of the disease 15, 16. OA is now increasingly regarded not only as a degenerative joint disorder but also as a metabolic disease, in which inflammatory signals induce a pathological shift from oxidative phosphorylation to glycolysis. This reprogramming enhances mTOR activity and ROS generation, creating a vicious cycle that accelerates cartilage degeneration. Rapamycin, an inhibitor of the mTOR subcomplex mTORC1, reduces the levels of ADAMTS5 and IL-1β and activates autophagy, thereby alleviating disease severity in mouse models of OA 13, 17. These observations highlight metabolic pathways and their regulators as potential therapeutic targets in OA.

Recent literature further emphasizes the importance of metabolic reprogramming in OA pathogenesis, framing it as a central driver of cartilage degeneration and immune dysregulation 13, 18, 19. Nevertheless, therapeutic approaches specifically aimed at restoring mitochondrial function and correcting metabolic imbalance in OA remain limited. While our previous study introduced MSC-Au-Steroid in the context of RA with a primary focus on inflammation suppression 12, chondrocyte-centered metabolic mechanisms were not investigated. Building upon these insights, the current work uniquely addresses this mechanistic and translational gap by evaluating whether MSC-Au-Steroid can restore chondrocyte mitochondrial metabolism and reprogram immune-metabolic interactions in OA.

Based on this rationale, we hypothesized that MSC-Au-Steroid could function not only as an anti-inflammatory agent but also as a metabolic modulator directly targeting mitochondrial dysfunction, ROS overproduction, and mTOR hyperactivation. We postulated that by preserving mitochondrial integrity and reducing oxidative stress, MSC-Au-Steroid would restore metabolic homeostasis in chondrocytes and promote cartilage repair. This dual mechanism formed the conceptual basis of our study. MSCs provide immunomodulatory support that can influence cellular metabolism, while Au-conjugated steroid enhances local anti-inflammatory effects. Together, these features were expected to alleviate mitochondrial dysfunction, suppress ROS, and modulate mTOR signaling in OA chondrocytes. In this study, we investigated whether MSC-Au-Steroid could modulate key metabolism-related pathways in articular cartilage from OA patients and animal models to evaluate its potential as a DMOAD. MSC-Au-Steroid interacted with activated mTOR in chondrocytes through the DDIT4 network and regulated excessive ROS production and the overexpression of MMPs and ADAMTS caused by mitochondrial dysfunction. The two components of MSC-Au-Steroid-MSCs and Au-conjugated steroid-acted synergistically to promote cartilage repair, restore cartilage-regeneration factors, regulate immune responses, and rebalance T cell subsets. Collectively, MSC-Au-Steroid, a nano-based therapeutic system, modulated metabolic mechanisms within chondrocytes and demonstrated the effectiveness of a multifactor strategy for treating OA.

## Materials and Methods

### Anti-CD90-Au-Steroid conjugates

Au-CD90-Steroid conjugates were synthesized according to a previously reported method 12. Briefly, Au-Steroid complexes were synthesized through covalent conjugation of anti-CD90 Abs to PEGylated star-shaped gold nanoparticles (Au), followed by non-covalent attachment of triamcinolone (Steroid). Anti-CD90 antibodies were conjugated to Au using N-Hydroxysuccinimide (NHS)-1-ethyl-3-(dimethylaminopropyl) carbodiimide hydrochloride (EDC) in an EDC/NHS coupling reaction to form Au-CD90, which was then conjugated with TA to produce Au-CD90-Steroid. MSC were generated by incubating naïve MSC in conditioned medium collected from LPS-stimulated J774A.1 macrophages and TNF-α-stimulated human FLSs. J774A.1 cells (1 × 10^6^) were treated with 50 ng mL^-1^ LPS in DMEM, and the conditioned medium was harvested after 24 h. Similarly, FLSs (5 × 10^5^) were treated with 20 ng mL^-1^ recombinant TNF-α in DMEM, and conditioned medium was collected after 24 h. The conditioned medium from J774A.1 cells and FLSs was mixed in a 1:1 ratio to mimic an inflamed joint environment. MSC-Au-Steroid was synthesized by conjugating Au-CD90-Steroid to the membrane of MSC.

### Physicochemical characterization

The hydrodynamic size distribution and electrical potential of the nanodrugs, including Au-Steroid, were measured using Litesizer 500 (Anton Paar) following the manufacturer's instructions. The quantification of Au and non-covalently attached Steroid on Au was performed via absorbance measurements using a UV-vis spectrophotometer (Biochrom). The characteristic absorbance peak of Au was observed at 825 nm and was used to calculate its concentration. Steroid conjugated to Au exhibited an absorbance peak at approximately 242 nm, and the baseline absorbance of Au was subtracted to ensure accurate quantification of Steroid. In MSC-conjugated complexes, the absorbance peak of Steroid overlapped spectrally with that of Steroid at 242 nm. The amount of Steroid in the conjugated complex was quantified using two approaches. For the indirect method, the concentration of Au bound to MSCs was determined using UV-vis spectroscopy, and the quantification of TA in MSC-Au-Steroid was based on the evaluation of Au binding. This process was performed using a standard curve generated from absorbance measurements at 810 nm (Figure S1A). For the direct method, MSC-Au-Steroid complexes (1×10^6^ cells) were lyophilized and dissolved in 100 μL of methanol (34860; Sigma). The samples were then diluted with an ACN:DW = 50:50 solution prepared using acetonitrile (34881; Sigma), and analyzed using UPLC (ACQUITY UPLC H-Class PLUS System; Waters) equipped with an C18 column (ACQUITY UPLC BEH C18 column 1.7 μm; Waters) at 50 °C. The mobile phase conditions were as follows: an initial equilibration with 100% DW for 12 min, followed by a linear gradient to 32% ACN over 12 min. The system was then maintained at 32% ACN and 68% DW for 6 min. Subsequently, a gradient to 70% ACN was applied over 10 min and maintained for 2 min, before returning to 100% DW for 5 min to re-equilibrate the system. The TA peak was detected at 241 nm with a retention time (RT) of 28-32 min (Figure S1D). All samples and mobile phases were filtered through a 0.2 μm PTFE-D filter prior to injection. Further structural characterization of the morphologies, crystallinities, and elemental distributions of the Au-PEG and Au-Steroid complexes was conducted using TEM (F30, Tecnai, FEI-Philips, USA) and STEM (JEM-ARM200F, JEOL, Japan). EDS mapping was also performed to provide detailed insights into the elemental composition of the nanodrugs, confirming their successful conjugation and structural integrity.

### Isolation of chondrocytes and PBMCs

Primary chondrocytes and peripheral blood samples were obtained from patients with OA, aged 63-80 years, who were treated at Jeonbuk National University Hospital. The study protocol was reviewed and approved by the Institutional Review Board of Jeonbuk National University Hospital (IRB No. 2021-02-010-005), and written informed consent was obtained from all participants prior to the collection of samples. Cartilage tissues were finely minced in serum-free Dulbecco's Modified Eagle Medium (DMEM; LM001-05, Gibco) containing hyaluronidase (H3506; Sigma) and incubated at 36 °C. The tissue fragments were washed and digested with protease in serum-free DMEM for 1 h, followed by a second digestion in serum-free DMEM containing hyaluronidase and collagenase (C9263; Sigma) for 3 h. After enzymatic digestion, the resulting suspension was filtered through a cell strainer to isolate chondrocytes, which were cultured in DMEM supplemented with 10% heat-inactivated fetal bovine serum (#12483020; Gibco) and 1% penicillin-streptomycin (15140-122; Gibco) at 37 °C in a humidified 5% CO₂ incubator. Peripheral blood mononuclear cells (PBMCs) were isolated from whole blood using Ficoll-Paque PLUS (17144003; Cytiva) density-gradient centrifugation. The blood was diluted 1:1 with phosphate-buffered saline, layered onto the Ficoll solution, and centrifuged to separate the layers. The PBMC fraction was collected and washed for subsequent analyses.

### Cell culture and stimulation

Chondrocytes obtained from patients with degenerative OA were cultured in DMEM supplemented with 10% heat-inactivated FBS and 1% penicillin at 37 °C in a humidified atmosphere containing 5% CO₂. For stimulation, cells were treated with 1 × 10⁴ MSCs, 500 ng mL⁻¹ Au-Steroid, or MSC-Au-Steroid conjugates for 24 h in the presence of 10 ng mL⁻¹ recombinant IL-1β (200-01B, PeproTech). The clinical characteristics of the patients are summarized in Table S2. For T-cell activation, cells were stimulated with 1 μg mL⁻¹ anti-CD3 (555336, BD Biosciences) together with 500 μg mL⁻¹ Mito-TEMPO (SML0737, Sigma), Au-Steroid, MSC-Au-Steroid conjugates, or control conditions, and cultured for 72 h in RPMI medium containing 10% heat-inactivated FBS, 1% penicillin, 2 mM L-glutamine, 0.1 mM non-essential amino acids, and 100 mM sodium pyruvate.

### Flow cytometry

To verify the conjugation of Au-Steroid with adipose-derived mesenchymal stem cells (ADMSC), Au-Steroid was non-covalently linked to Alexa Fluor 488 (S11223; Invitrogen) according to a previously established protocol. The fluorescently labeled samples were analyzed using a BD LSR II flow cytometer (BD Biosciences), and data acquisition and analysis were performed with FlowJo software (version 10.1; FlowJo, LLC). For intracellular cytokine staining and T-cell subset profiling, cells were isolated from the joint tissues of MIA-induced mice and stimulated for 4 h with phorbol 12-myristate 13-acetate (PMA, 20 ng mL⁻¹; P8139, Sigma), ionomycin (1 μg mL⁻¹; I0634, Sigma), and Golgi plug (555028; BD Biosciences). The following antibodies were used for staining: anti-mouse CD4 (100544; BioLegend), IFN-γ (505806; BioLegend), IL-17A (560666; BD Biosciences), Foxp3 (25-5773-82; Invitrogen), F4/80 (123110; BioLegend), and CD163 (155319; BioLegend). Human PBMCs were stimulated under similar conditions using 50 ng mL⁻¹ PMA, 1 μg mL⁻¹ ionomycin, and Golgi plug for 4 h, followed by staining with anti-human CD4 (344632; BioLegend), CD73 (561558; BD Biosciences), IFN-γ (560742; BD Biosciences), TNF-α (557647; BD Biosciences), IL-17A (560490; BD Biosciences), Foxp3 (11-4776-42; Invitrogen), and RORγt (12-6988-82; Invitrogen). All samples were sequentially gated on singlets, then on FSC-A/SSC-A to remove debris, and subsequently on live cells identified using fixable viability dyes. Data acquisition was performed using an Attune NxT flow cytometer (Thermo Fisher Scientific), and analysis was carried out with FlowJo software (version 10.7.1).

### Animals

Ten-week-old male C57BL/6J mice were obtained from Nara Biotech and maintained under controlled environmental conditions (temperature: 23 ± 2 °C; relative humidity: 50%). All animal procedures were performed in accordance with the ethical standards and regulations of the Institutional Animal Care and Use Committee of Jeonbuk National University Medical School (approval number: NON2024-045).

### MIA-induced mouse model of OA

The MIA mouse model was generated by preparing a solution of sodium iodoacetate (I2512, Sigma) in 0.9% sodium chloride solution (7726-1400, Daejung) and injecting 0.75 mg 10 μL^-1^ into the mouse joint cavity and inducing for 28 days. MSC (1 × 10^6^ cells), Au-Steroid (3 mg/kg), and conjugated MSC (1 × 10^6^ cells)-Au-Steroid (3 mg/kg) were injected intraperitoneally at the same dose once a week. The mice were divided into five groups. Representative data are from three independent experiments, with 12 mice per group (4 mice per group in each experiment). On day 28, the mice were euthanized by CO_2_ inhalation, and joint tissues were collected. Some experimental groups were fixed in 4% formalin for pathological examination and micro-CT analyses, and some joints were stored at -80 °C for qPCR and western blot analyses. For FACS and Seahorse analyses, some joints from the groups were collected and treated with collagenase D (11088858001, Sigma-Aldrich) to dissociate single cells.

### Histological analysis

Knee joint tissues were fixed in 4% formalin, embedded in paraffin, and sectioned at a thickness of 4 μm. The sections were stained with hematoxylin and eosin (H&E) as well as safranin O for histological evaluation. Synovitis severity was assessed from H&E-stained sections under ×200 magnification and scored on a scale of 0-9: scores of 0-1 indicated no synovitis, 2-4 represented mild synovitis, and 5-9 denoted severe synovitis. Cartilage degeneration was graded according to the OARSI scoring system: grade 0, normal cartilage; grade 1, superficial fibrillation; grade 2, focal surface discontinuity; grade 3, vertical fissuring; grade 4, erosion of the cartilage layer; grade 5, subchondral bone involvement with cartilage loss; and grade 6, complete cartilage deformation. Osteophyte maturity was evaluated on a 0-3 point scale, where 0 indicated absence, 1 represented predominantly cartilaginous osteophytes, 2 reflected active vascular invasion and endochondral ossification, and 3 denoted mature bone formation. Subchondral bone plate (SBP) thickness was quantified from safranin O-stained images at ×200 magnification by measuring five randomly selected regions per sample.

### Western blotting

Cell lysates and western blots were analyzed using SDS-PAGE as previously described 20. Membranes were incubated with the following primary antibodies: anti-AMPKα (1:1000, #2532; Cell Signaling), anti-phospho-AMPKα (1:1000, #2535; Cell Signaling), anti-DDIT4 (1:3000, 10638-1-AP; Proteintech), anti-phospho-mTOR (1:1000, #5536; Cell Signaling), anti-mTOR (1:1000, #2983; Cell Signaling), anti-phospho-Raptor (1:1000, #2083; Cell Signaling), anti-Raptor (1:1000, #2280; Cell Signaling), anti-phospho-p70S6K (1:1000, #9205; Cell Signaling), anti-p70S6K (1:1000, #9202; Cell Signaling), anti-phospho-4EBP1 (1:1000, #9451; Cell Signaling), anti-4EBP1 (1:1000, #9452; Cell Signaling), anti-MMP-1 (1:1000, #PA5-27210; Invitrogen), anti-MMP-3 (1:500, #PA5-119639; Invitrogen), anti-MMP-9 (1:2000, #PA5-13199; Invitrogen), anti-MMP-13 (1:1000, #PA5-27242; Invitrogen), and anti-β-actin (1:1000, #4970; Cell Signaling). Goat anti-rabbit IgG (polyclonal, 1:10000; ADI-SAB-300, Enzo Life Sciences) was used as the secondary antibody, and immunoreactive bands were visualized using an enhanced chemiluminescence substrate (ECL, 34580; Thermo Fisher Scientific). Protein signals were captured using an Amersham Imager 600 system (Cytiva Life Sciences, GE Healthcare).

### Micro-computed tomography

The right femurs and tibias of the mice were dissected, fixed in 4% paraformaldehyde (J19943-K2, Thermo Fisher), and washed with PBS; images were then acquired using a high-resolution micro-CT system (SkyScan 1276; SkyScan, USA) at 68 mm, with a source voltage of 6 kVp, a current of 57 μA, and 300 ms per frame. Data were analyzed using CTAn and CTvox software (Bruker).

### Metabolic flux assays

Human chondrocytes and mouse joint-derived cells were seeded at a density of 5 × 10⁵ cells per well on poly-D-lysine-coated (A3890401; Gibco) XF miniplates. Cells were equilibrated for 1 h prior to the assay in XF DMEM or XF RPMI medium containing 10 mM glucose, 2 mM glutamine, and 1 mM pyruvate. The extracellular acidification rate (ECAR; 103020-100, Agilent) and oxygen consumption rate (OCR; 103015-100, Agilent) were measured using a Seahorse XFe24 analyzer (Agilent) in accordance with the manufacturer's instructions.

### Mitochondrial content measurement

Mouse joint cells, human chondrocytes, and PBMCs isolated from patients with OA were plated in 96-well plates at a density of 5 × 10⁵ cells per well for mitochondrial content analysis. The cells were incubated at 37 °C for 1 h with MitoTracker Green FM (M46750; Thermo Fisher), MitoSOX Red (M36008; Thermo Fisher), and MitoCMXRos (M46752; Thermo Fisher). Prior to flow cytometric analysis, cells were stained with a Live/Dead Fixable Dead Cell dye (65-0865-14; Invitrogen) to exclude non-viable cells. Fluorescence intensity was quantified using an Attune NxT flow cytometer (Thermo Fisher Scientific). The measurement of relative mtDNA copy number was performed by qPCR following methods previously described 21.

### Glucose uptake

Human primary chondrocytes (2 × 10⁴ cells per well) were cultured in 96-well plates with DMEM and stimulated for 24 h with recombinant IL-1β, MSCs, Au-Steroid, or MSC-Au-Steroid conjugates. In parallel, mouse joint-derived cells collected on day 28 were incubated with 2-[N-(7-nitrobenz-2-oxa-1,3-diazol-4-yl)amino]-2-deoxy-D-glucose (2-NBDG, 0.01 mg mL⁻¹; Thermo Fisher) at 37 °C for 30 min to assess glucose uptake. Prior to flow cytometric analysis, all samples were stained with Live/Dead Fixable Dead Cell dye (65-0865-14; Invitrogen) to exclude non-viable cells. Data acquisition was performed using an Attune NxT flow cytometer (Thermo Fisher Scientific).

### RNA isolation and qPCR

Total RNA was isolated from frozen tissue samples and cultured cells using RNAiso Plus (Takara, Tokyo, Japan) in accordance with the manufacturer's instructions. Complementary DNA (cDNA) was synthesized and subjected to quantitative PCR (qPCR) as described previously, following the same manufacturer's protocol. For amplification, cDNA templates were combined with SYBR Green Master Mix and gene-specific primers. Relative mRNA expression levels were calculated using the 2⁻ΔΔCt method, with β-actin serving as the internal reference gene. All reactions were performed on a QuantStudio™ 5 Real-Time PCR System (Applied Biosystems™), and the primer sequences are listed in Table S3.

### siRNA transfection

Chondrocytes were transfected with siRNAs targeting DDIT4 (sc-45806; Santa Cruz Biotechnology) or AMPKα1/2 (sc-45312; Santa Cruz Biotechnology), along with a non-targeting control siRNA (sc-37007; Santa Cruz Biotechnology), using FuGENE® SI reagent according to the manufacturer's instructions. After 48 h of transfection, cells were stimulated for 24 h with recombinant IL-1β, MSCs, Au-Steroid, or MSC-Au-Steroid conjugates.

### Confocal microscopy

To examine the conjugation of Au-Steroid with adipose-derived mesenchymal stem cells (ADMSC), cells were cultured on poly-D-lysine-coated coverslips. Alexa Fluor 488-labeled Au-Steroid was coupled to ADMSC according to a previously established protocol. The resulting MSC-Au-Steroid conjugates were rinsed twice with PBS and fixed overnight in 2% paraformaldehyde at 4 °C. Fixed cells were then blocked with 1% bovine serum albumin (BSA) for 2 h at room temperature. To visualize the Au-Steroid-conjugated anti-CD90 antibodies, samples were incubated with goat anti-rabbit IgG H&L (Texas Red) (ab6719; Abcam) for 2 h at 37 °C. ADMSC cytoskeletal structures were labeled with rhodamine-conjugated phalloidin, and coverslips were mounted using an antifade mounting medium (H-1600-10; Vector Laboratories). For assessment of mitochondrial function in human chondrocytes, 2 × 10⁵ cells per well were seeded in 8-well chamber slides (PEZGS0816; Millicell® EZ Slide) and treated with IL-1β, MSCs, Au-Steroid, or MSC-Au-Steroid for 24 h. MitoTracker Green FM and MitoSOX Red (Thermo Fisher) were added, and cells were incubated at 37 °C for 1 h. Afterward, cells were fixed in 4% paraformaldehyde for 15 min at 4 °C, permeabilized with 0.3% Triton X-100 (T8787; Sigma), and stained with DAPI (1 µg mL⁻¹; 62248, Thermo Fisher) for 5 min at room temperature. Fluorescence images were captured using an LSM 980 confocal microscope (Carl Zeiss Microscopy, Germany).

### Statistical analysis

Statistical analyses were conducted using GraphPad Prism software (version 10.0; GraphPad Software, San Diego, CA, USA). Differences among experimental groups were assessed by one-way analysis of variance (ANOVA) followed by the Holm-Šídák multiple-comparison test. Data are expressed as the mean ± standard error of the mean (SEM). Statistical significance was defined as p < 0.05, *p < 0.01, **p < 0.001, and ***p < 0.0001.

## Results and Discussion

### Physical properties of synthesized nanodrugs on the membrane of educated MSC

**Figure 1:**
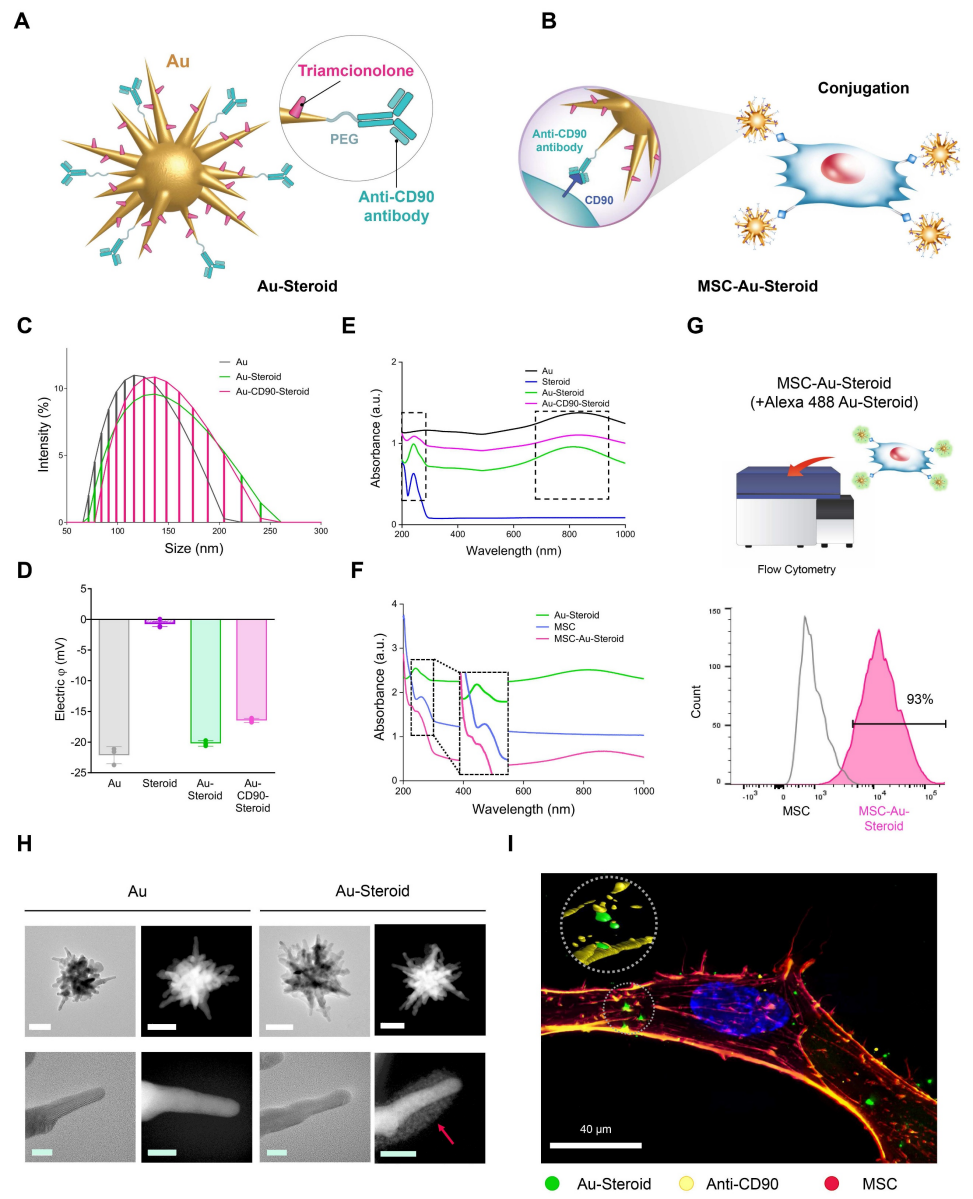
** Physicochemical properties of the gold nanostar (Au)-triamcinolone (Steroid) with MSC. (A)** Illustration of chemical bonding of gold nanostar (Au)-triamcinolone (Steroid). PEGylated Au (Au-PEG) non-covalently conjugated to Steroid. **(B)** Illustration of MSC and Au-CD90-Steroid conjugation using CD90 and anti-CD90 antibodies (Abs). **(C)** Hydrodynamic size distribution of Au-PEG, Au-Steroid and Au-CD90-Steroid. **(D)** Mean hydrodynamic sizes and electrical potentials of PEGylated Au (Au-PEG), Au-Steroid, AuS-C0-TA. **(E)** Ultraviolet-visible spectroscopy (UV-vis) analysis of Steroid peak at 242 nm and AuS peak at 810 nm, verifying the formation of Au-Steroid. **(F)** UV-vis analysis of Steroid peak at 242 nm, MSC peak at 260 nm, and Au peak at 810 nm, with the MSC-Au-Steroid conjugate showing shifted peak at 230 nm (shifted from 242 nm peak of MSC) and 250 nm (shifted from 260 nm peak of Au-Steroid), indicating conjugation. **(G)** Flow cytometry analysis of MSC-Au-Steroid confirms conjugation of Au-Steroid by surface-bound by Alexa 488-labeled Au-Steroid and Alex 488-labeled secondary antibodies targeting anti-CD90 antibodies. The percentage of Alexa488 fluorescence intensity of MSC and MSC-Au-Steroid, as analyzed in Fig. 1g. Data represent mean ± SEM (*n* = 3). **(H)** Transmission electron microscopy (TEM) images of Au and Au-Steroid. Scale bar (white) = 100 nm, (green) = 10 nm. **(I)** Confocal microscopy analysis of Au-Steroid (green), anti-CD90 Ab (yellow), and MSC (red). Scale bar= 20μm. Inset image indicate surface localization of Au-Steroid on MSC, as confirmed by 3D analysis of conjugation via anti-CD90 Ab.

MSC was pretreated with conditioned medium from TNF-α-stimulated fibroblast-like synoviocytes (FLSs) and Lipopolysaccharides (LPS)-stimulated J774 macrophages, inflammatory cytokine treated were employed in this study as therapeutic vectors to enhance migratory capacity toward inflammatory cells 12. PEGylated star-shaped gold nanoparticles (Au) were synthesized and conjugated with triamcinolone (Steroid) to treat inflammation. Anti-CD90 antibodies (Abs) were covalently conjugated to Au-Steroid for targeting CD90, a membrane protein highly expressed on MSC. This was designed to facilitate the conjugation of Au-Steroid to MSC membranes. The primary procedure involved the covalent conjugation of anti-CD90 Abs to Au, followed by the non-covalent conjugation of Steroid (Figure 1A). The resulting Au-CD90-Steroid complex was conjugated with MSC to produce MSC-Au-Steroid (Figure 1B). The amount of Steroid attached to Au was quantified using a standard absorbance curve at 810 nm (Figure S1), and the Au ratio was calculated as 1:2.39 (± 0.42) (Table S1). This optimized conjugation ratio was identical to that reported in our previous study 12. Detailed experimental values supporting this ratio are available in Supplementary Tables 1-3 of the same study 12. The hydrodynamic sizes of Au-PEG, Au-Steroid, and Au-CD90-Steroid (Au-Steroid conjugated with CD90) for binding to MSC were 132, 143, and 150 nm, respectively (Figure 1C). Surface charge analysis showed that free Steroid exhibited a nearly neutral charge, whereas Au-PEG, Au-Steroid, and Au-CD90-Steroid were negatively charged (Figure 1D). The steroid loading in MSC-Au-Steroid was assessed by both UV-vis and UPLC analysis. UV-vis spectroscopy revealed absorbance peaks at 810 nm for Au, 260 nm for MSC, and 242 nm for Steroid in Au-Steroid and MSC-Au-Steroid (Figure 1E-F). A slight shift in the absorbance peak after conjugation indicated effective binding (Figure 1F). Quantitatively, UV-vis analysis yielded a steroid loading of 246 µg (±30.00), while UPLC analysis yielded 241.13 µg (±0.82), demonstrating a high level of consistency between the two methods (Figure S1D and Table S1). Fluorescence-activated cell sorting (FACS) further confirmed the conjugation of MSC-Au-Steroid. Surface-bound Au-Steroid was detected using Alexa 488-labeled Au-Steroid and Alexa 488-conjugated secondary antibodies targeting anti-CD90, both emitting green fluorescence. Further analysis revealed that most MSC-Au-Steroid complexes exhibited distinct fluorescence, confirming efficient conjugation. (Figure 1G). Transmission electron microscopy (TEM) and scanning TEM (STEM) confirmed the star-shaped morphology of the PEGylated Au-Steroid complex, with additional structures covering the branches of Au (Figure 1H). Energy-dispersive X-ray spectroscopy (EDS) showed signals from Au, S (sulfur: PEG), and F (fluorine: TA) at identical locations (Figure S1). Confocal microscopy images confirmed the successful conjugation of Au-Steroid to the MSC membrane (Figure 1I), verifying the effective formation of MSC-Au-Steroid complexes. In addition, the drug release profile of MSC-Au-Steroid was analyzed. Under 4°C conditions, 13.6% of the loaded steroid was released within 6 hours. These results further support the stability of MSC-Au-Steroid and demonstrate that premature drug release is minimal, thereby validating the sustained-release property of the system. Compared to AuS-Steroid, MSC-AuS-Steroid exhibited enhanced targeting efficiency and improved drug delivery, driven by the synergistic effects of MSC-mediated inflammation homing and the sustained release of steroid. Collectively, these findings suggest that the MSC-Au-Steroid offers greater therapeutic potential by combining the targeted migratory capacity of MSC with the anti-inflammatory properties of steroid.

### MSC-Au-Steroid regulate the abnormal metabolic program in the inflammatory environment of OA chondrocytes

**Figure 2:**
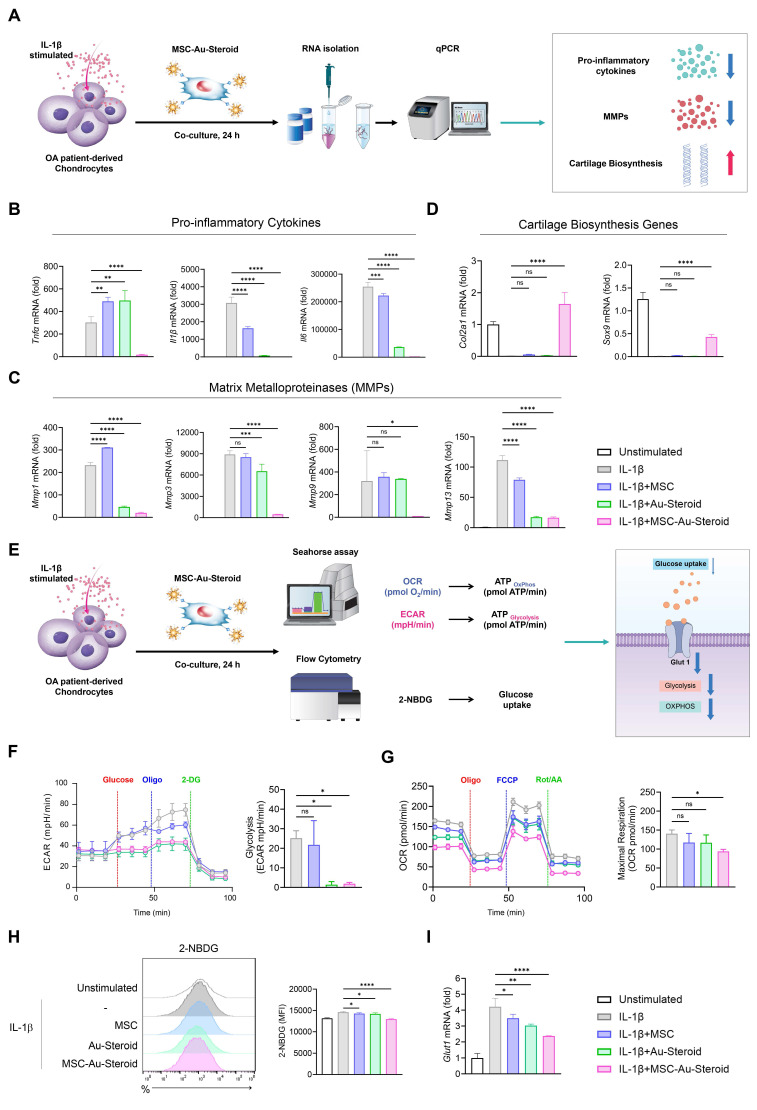
** MSC-Au-Steroid regulates osteoarthritis-related genes and metabolic changes in IL-1β-stimulated OA chondrocytes. (A)** Schematic illustration of the qPCR analysis workflow in OA chondrocytes treated with MSC-Au-Steroid. **(B-I)** OA chondrocytes were stimulated with IL-1β (10 ng mL⁻¹) for 24 h in the presence or absence of MSCs (1 × 10⁴ cells), Au-Steroid (500 ng mL⁻¹), or MSC-Au-Steroid conjugates (1 × 10⁴ cells and 500 ng mL⁻¹) (n = 3). **(B-D)** Relative mRNA expression of pro-inflammatory cytokines (*Tnfα*, *Il1b*, and *Il6*), cartilage matrix-related genes (*Col2a1*, *Sox9*), and matrix-degrading enzymes (*Mmp1*, *Mmp3*, *Mmp9*, and *Mmp13*) was quantified by qPCR. **(E)** Schematic diagram showing the metabolic analysis of OA chondrocytes treated with MSC-Au-Steroid, including assessments of oxygen consumption rate (OCR), extracellular acidification rate (ECAR), and glucose uptake. **(F)** Real-time ECAR profiles of OA chondrocytes in response to glucose, oligomycin, and 2-DG, with the corresponding bar graph showing glycolytic activity. **(G)** Real-time OCR traces of OA chondrocytes upon treatment with oligomycin, FCCP, and Rot/AA, and quantification of maximal respiratory capacity. **(H)** Representative flow cytometry histograms (left) and bar graphs (right) illustrating glucose uptake in OA chondrocytes after incubation with 2-NBDG for 2 h. **(I)** Expression of *Glut1* analyzed by qPCR. Data are presented as mean ± SEM. ****p < 0.0001, ***p < 0.001, **p < 0.01, *p < 0.05.

In OA, inflammatory cytokines such as TNF-α, IL-1β, and IL-6 induce metabolic changes through the upregulation of degradative enzymes such as MMPs and downregulation of chondroanabolic markers such as COL2A1 and SOX9 22. In this inflammatory environment, the abnormal energy metabolism of chondrocytes is converted to glycolysis, which promotes cartilage degeneration and OA development 22-26. Accordingly, we cultured chondrocytes from the recruited patients with OA and stimulated them with IL-1β to investigate the protective effects of MSC-Au-Steroid on chondrocytes (Figure 2A). MSC-Au-Steroid inhibited IL-1β-stimulated inflammatory cytokines (*Tnfα*, *Il1β*, and *Il6*) and the gene and protein expression of MMP*s* (*Mmp1*, *Mmp3*, *Mmp9*, and *Mmp13*) in OA chondrocytes (Figure 2B-C; Figure S2A-B). Furthermore, compared to that with MSC and Au-Steroid, the expression of *Col2a1* and *Sox9* was significantly increased with MSC-Au-Steroid, indicating that chondrogenic activity was maintained even in an inflammatory environment (Figure 2D).

After IL-1β stimulation, OA chondrocytes exhibited an altered metabolic profile related to glucose transport regulation and its corresponding actions 13. We thus analyzed the extracellular acidification rate (ECAR) and mitochondrial oxygen consumption rate (OCR) under inflammatory conditions to measure glycolysis and oxidative phosphorylation (OXPHOS), respectively, in OA chondrocytes (Figure 2E). IL-1β stimulation increased glycolysis, glycolytic capacity, and glycolytic reserves compared to that in the unstimulated group, and these values were further reduced by MSC-Au-Steroid treatment compared to those with MSC and Au-Steroid treatment (Figure 2F; Figure S2C). Furthermore, when assessing the functional profile of mitochondria by measuring the OCR during sequential treatment with oligomycin (an ATP synthase inhibitor), FCCP (an H^+^ ion transporter), and rotenone (an electron transport chain inhibitor), IL-1β stimulation significantly increased basal respiration, maximal respiration, and ATP levels compared to those in the unstimulated group, whereas MSC-Au-Steroid treatment reliably downregulated these values (Figure 2G; Figure S2D). Interestingly, the efficacy of the MSC-Au-Steroid conjugate was greater than that of co-treatment with MSC and Au-Steroid (Figure S2E-H).

Glucose uptake is the first rate-limiting step in glucose metabolism 27. To determine whether the downregulation of glycolysis induced by MSC-Au-Steroid resulted from a decrease in glycolytic flow, we measured the intracellular glucose uptake by FACS using the fluorescent glucose analog 2-NBDG. IL-1β stimulation increased glucose uptake, but compared to that with MSC and Au-Steroid treatment, MSC-Au-Steroid treatment showed the most significantly inhibited glucose uptake (Figure 2H). Glucose transporter 1 (GLUT1) facilitates glucose transport across the chondrocyte membrane and is essential for chondrogenesis 22. Although *Glut1* expression is increased by pro-inflammatory cytokines, normal chondrocytes maintain normal cartilage development by regulating *Glut1* expression to adapt to changes in extracellular glucose concentration across the cell membrane 28. Indeed, our OA chondrocyte data showed that, although the gene expression of Glut1 was increased by IL-1β, it was most substantially decreased by the MSC-Au-Steroid treatment (Figure 2I). These results suggest that the treatment with MSC-Au-Steroid inhibited the expression of inflammatory cytokines and MMPs in OA chondrocytes and effectively contributed to the up-regulation of chondrogenesis and changes in energy metabolism.

### MSC-Au-Steroid prevents the accumulation of dysfunctional mitochondria in OA chondrocytes

**Figure 3:**
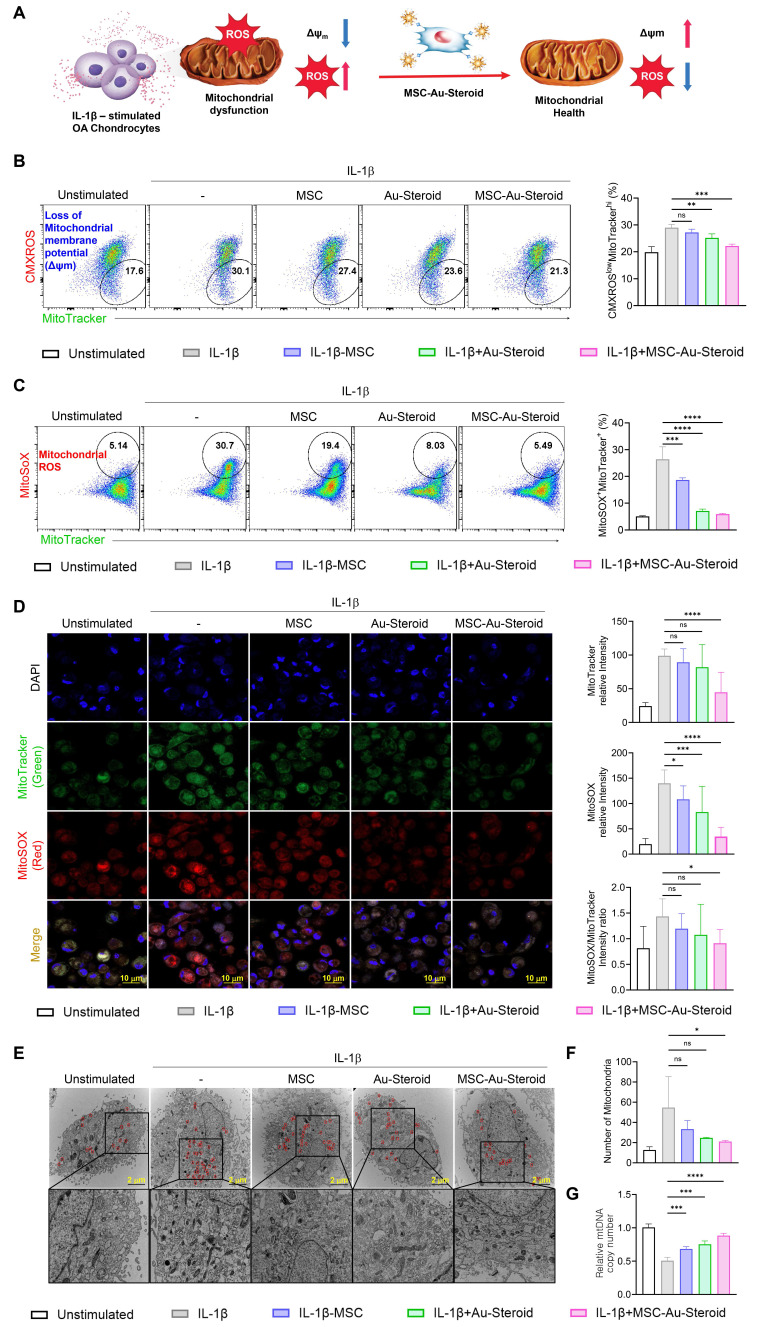
** MSC-Au-Steroid inhibits the accumulation of dysfunctional mitochondria and reduces the production of mitochondrial ROS. (A)** Schematic illustration of the regulatory mechanism of mitochondrial dysfunction by MSC-Au-Steroid. **(B-G)** OA chondrocytes were stimulated with IL-1β (10 ng mL⁻¹) for 24 h in the presence or absence of MSCs (1 × 10⁴ cells), Au-Steroid (500 ng mL⁻¹), or MSC-Au-Steroid conjugates (1 × 10⁴ cells and 500 ng mL⁻¹) (n = 3). **(B-C)** Representative flow cytometry plots (left panels) and quantification graphs (right panels) showing the proportion of dysfunctional mitochondria (CMXRos^low^MitoTracker Green^high^) with impaired membrane potential and mitochondrial ROS generation (MitoSOX⁺MitoTracker Green⁺). Mitochondrial membrane potential (Δψm) and mitochondrial ROS levels were determined using CMXRos and MitoSOX staining, respectively. **(D)** Representative fluorescence microscopy images showing mitochondrial mass (MitoTracker Green) and mitochondrial ROS production (MitoSOX Red) in OA chondrocytes (left panels), with corresponding quantification of fluorescence intensity (right panels). **(E)** Transmission electron microscopy (TEM) images depicting mitochondrial morphology in OA chondrocytes under the indicated conditions. Mitochondria are indicated by red arrowheads (scale bar: 2 μm). **(F)** Quantification of mitochondrial number per cell. **(G)** Relative mitochondrial DNA (mtDNA) copy number determined by qPCR. Data are expressed as mean ± SEM. ****p < 0.0001, ***p < 0.001, **p < 0.01, *p < 0.05.

In OA, reactive oxygen species (ROS) production and oxidative damage occur in excess because of structural and functional changes in the mitochondria. This leads to impaired chondrocyte function and enhanced extracellular matrix degradation, apoptosis, and joint cartilage destruction, thereby accelerating OA progression 13. We investigated whether the inflammatory environment of OA chondrocytes, as described in Figure 2, was derived from abnormal mitochondrial function and metabolic profile and whether MSC-Au-Steroid effectively regulated this condition (Figure 3A).

First, we measured the total mitochondrial content regardless of mitochondrial membrane potential (Δψ_m_) by staining the cells with MitoTracker Green. We found that mitochondrial mass increased in OA chondrocytes after IL-1β stimulation. In contrast, MSC-Au-Steroid treatment reduced mitochondrial mass (Figure S3A). Additionally, to evaluate ROS levels in mitochondria, we used MitoSOX, a mitochondria-specific ROS indicator, to detect the presence of superoxide ions in live cells through flow cytometry. MSC-Au-Steroid treatment significantly reduced ROS more effectively than did MSC alone and showed inhibition levels similar to those with Au-Steroid treatment (Figure S3B).

Based on these results, we hypothesized that the mitochondrial mass in the inflammatory environment of OA chondrocytes increased due to the accumulation of dysfunctional mitochondria caused by the loss of Δψm, and that MSC-Au-Steroid inhibited this accumulation. To confirm this, we used MitoTracker Red (CMXROS; a Δψm-dependent mitochondrial dye) and MitoTracker Green (a Δψm-independent mitochondrial dye) together to distinguish between respiring and dysfunctional mitochondria. IL-1β stimulation led to an abnormal increase in dysfunctional mitochondria (CMXROS^low^, MitoTracker Green^high^) which was associated with enhanced ROS production. MSC-Au-Steroid treatment reversed this imbalance, significantly reducing mitochondrial ROS levels (MitoSOX^+^, MitoTracker Green^high^) and maintaining mitochondrial integrity (Figure 3B-C). Consequently, MSC-Au-Steroid inhibited the accumulation of dysfunctional mitochondria in the inflammatory environment of OA chondrocytes and significantly reduced mitochondrial ROS levels (MitoSOX^+^, MitoTracker Green^high^) (Figure 3B-C). Importantly, the observed changes in MitoTracker fluorescence intensities reflect the balance between healthy respiring and dysfunctional mitochondria. In IL-1β-stimulated OA chondrocytes, increased MitoTracker Green together with reduced MitoTracker Red signals indicate the accumulation of depolarized, ROS-producing mitochondria. MSC-Au-Steroid reversed this imbalance, thereby maintaining mitochondrial integrity and reducing oxidative stress. Live cell imaging further confirmed the accumulation of ROS-producing dysfunctional mitochondria under inflammatory conditions and their suppression by MSC-Au-Steroid (Figure 3D). To further validate these findings, we next performed transmission electron microscopy (TEM) and mtDNA copy number analysis. TEM images revealed that IL-1β stimulation induced swollen mitochondria with disrupted cristae and an abnormal increase in mitochondrial counts per cell, consistent with dysfunctional mitochondrial accumulation. In contrast, MSC-Au-Steroid treatment markedly reduced this abnormal accumulation and preserved mitochondrial structural integrity (Figure 3E-F). Complementarily, quantification of mtDNA copy number demonstrated that IL-1β increased mtDNA content, indicative of dysfunctional organelle accumulation, whereas MSC-Au-Steroid normalized mtDNA copy numbers (Figure 3G). Together, these results suggest that MSC-Au-Steroid effectively inhibited the accumulation of dysfunctional mitochondria in the inflammatory environment of OA chondrocytes and slowed OA progression by reducing mitochondria-derived ROS production.

### MSC-Au-Steroid inhibits the enhanced mTOR in OA chondrocytes and upregulates DDIT4

mTOR plays an important role in cell metabolism, growth, mitochondrial biosynthesis, and energy production, and is essential for cartilage development and growth 29. Additionally, mTOR expression is significantly increased in human OA cartilage and in experimental OA models 15. Inhibition of mTOR increases chondrocyte autophagy, which contributes to chondrocyte regeneration by removing cellular components damaged from OA 16.

**Figure 4:**
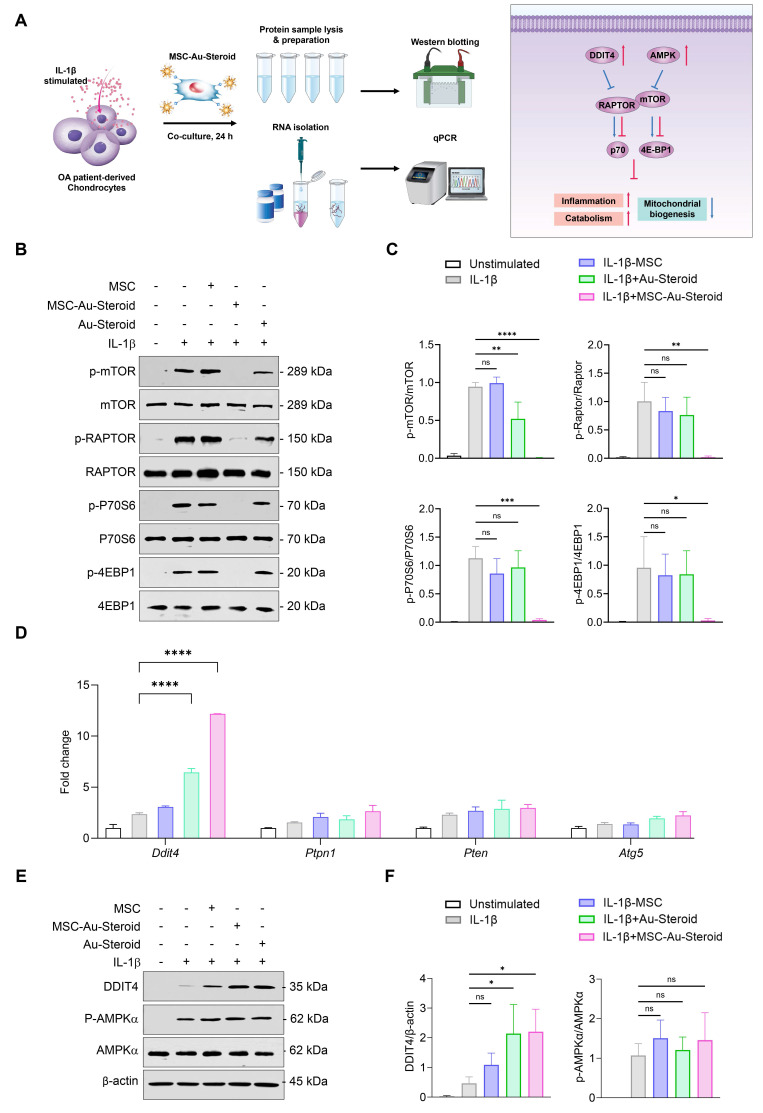
** MSC-Au-Steroid suppresses elevated mTOR activity in OA chondrocytes and promotes the expression of DDIT4. (A)** Schematic illustration depicting how MSC-Au-Steroid modulates the mTOR signaling pathway to attenuate inflammation in OA chondrocytes. **(B-F)** OA chondrocytes were stimulated with IL-1β (10 ng mL⁻¹) for 24 h in the presence or absence of MSCs (1 × 10⁴ cells), Au-Steroid (500 ng mL⁻¹), or MSC-Au-Steroid conjugates (1 × 10⁴ cells and 500 ng mL⁻¹) (n = 3). **(B)** Western blot analysis of mTOR and its downstream mTORC1 targets. **(C)** Quantification of relative protein band intensities. **(D)** Gene expression of mTOR-regulatory factors *(Ddit4*, *Ptpn1*, *Pten*, and *Atg5*) was analyzed by qPCR. **(E)** Western blot analysis of DDIT4 and AMPKα expression. **(F)** Quantification of corresponding protein band intensities. Data are presented as mean ± SEM. ****p < 0.0001, ***p < 0.001, **p < 0.01, *p < 0.05.

Considering the effects of MSC-Au-Steroid on mitochondria, we investigated whether MSC-Au-Steroid regulated the activity of mTOR and the mTORC1 (mTOR Complex 1) pathway, one of the two protein complexes of mTOR (Figure 4A). Upon stimulation of OA chondrocytes with IL-1β, activation of mTOR and mTORC1 was observed, as evidenced by increased phosphorylation of mTORC1 downstream target proteins such as RAPTOR, P70S6, and 4EBP1. However, in cells treated with MSC-Au-Steroid, the activation of mTOR and mTORC1 was significantly inhibited (Figure 4B-C). Notably, compared to cells treated with either MSC or Au-Steroid alone, MSC-Au-Steroid strongly downregulated mTOR and mTORC1 activity to baseline levels.

Therefore, we further examined how MSC-Au-Steroid inhibited mTOR signaling. Using qPCR analysis, we investigated whether MSC-Au-Steroid transcriptionally regulated metabolic pathways leading to mTOR signaling inhibition and found that among the *Ddit4*,* Ptpn1*,* Pten*, and* Atg5* genes, which can negatively regulate mTOR and mTORC1, *Ddit4* was strongly induced by MSC-Au-Steroid (Figure 4D). This induction was also confirmed at the protein level by western blotting (Figure 4E-F). MSC-Au-Steroid did not contribute to OA alleviation by restoring AMPK activity and inhibiting the mTOR pathway, unlike previous studies 5.

### DDIT4 induction by MSC-Au-Steroid inhibits mTOR signaling and maintains mitochondrial fitness

**Figure 5:**
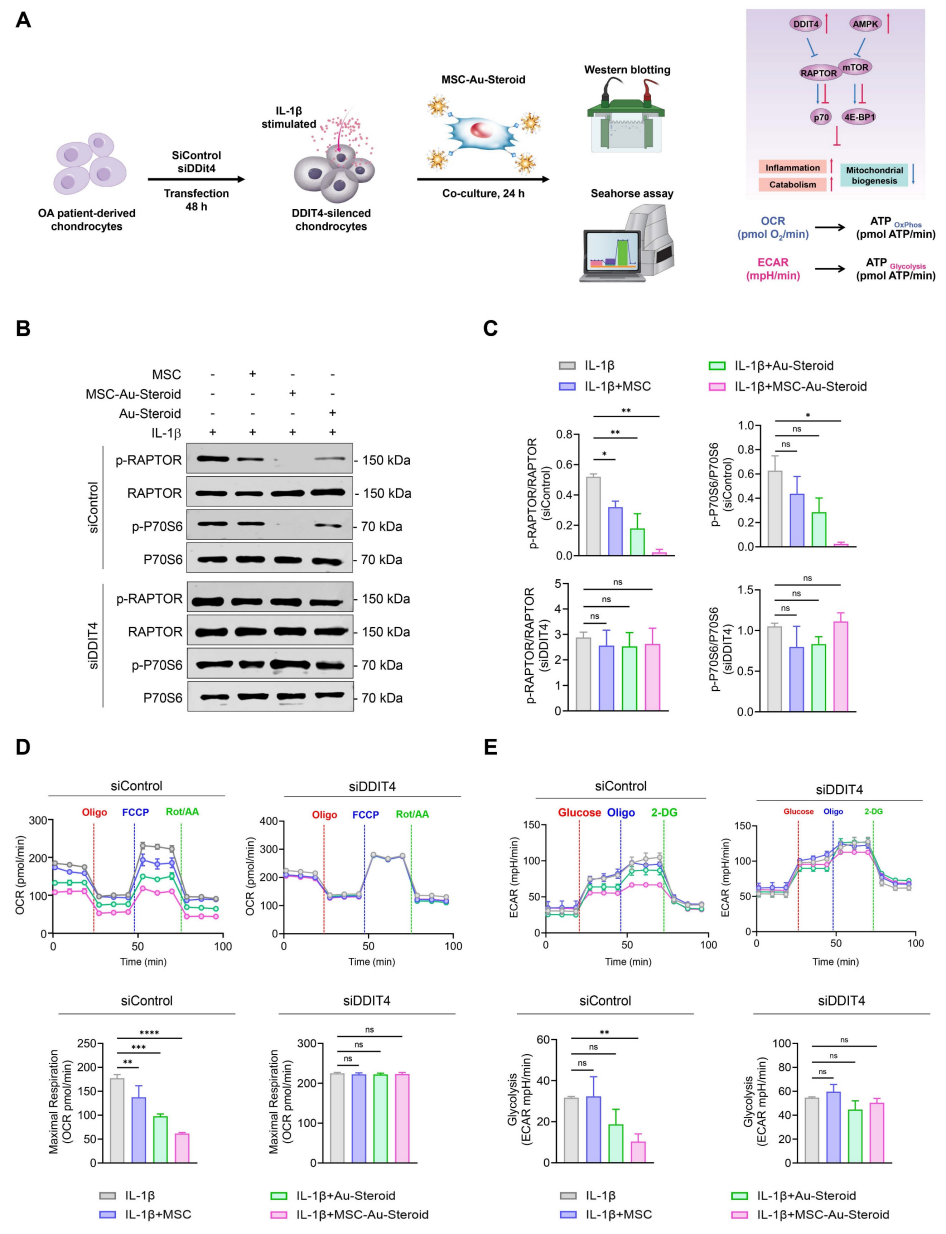
** MSC-Au-Steroid induces DDIT4, which suppresses mTOR signaling and supports mitochondrial health. (A-E)** OA chondrocytes were transfected with either control siRNA or DDIT4-specific siRNA for 48 h, followed by stimulation with IL-1β (10 ng mL⁻¹) in the presence or absence of MSCs (1 × 10⁴ cells), Au-Steroid (500 ng mL⁻¹), or MSC-Au-Steroid conjugates (1 × 10⁴ cells and 500 ng mL⁻¹) (n = 3). **(A)** Schematic representation of the experimental workflow illustrating siRNA transfection, subsequent treatment, and analysis in OA chondrocytes. **(B)** Western blot analysis of RAPTOR and P70S6 protein expression in cells transfected with control or DDIT4 siRNA. **(C)** Quantification of relative protein band intensities. **(D)** Real-time oxygen consumption rate (OCR) profiles of chondrocytes transfected with control or DDIT4 siRNA in response to oligomycin, FCCP, and Rot/AA (top panel), with bar graphs summarizing maximal respiratory capacity (bottom panel). **(E)** Real-time extracellular acidification rate (ECAR) profiles of OA chondrocytes transfected with control or DDIT4 siRNA following sequential injection of glucose, oligomycin, and 2-DG (top panel), and corresponding bar graphs showing glycolytic activity (bottom panel). Data are expressed as mean ± SEM. ****p < 0.0001, ***p < 0.001, **p < 0.01, *p < 0.05.

To evaluate the role of MSC-Au-Steroid-induced DDIT4, we transfected OA chondrocytes with siRNA to knock down *Ddit4* expression (Figure S3C). Following Ddit4 silencing, cells were treated with IL-1β in the presence or absence of MSCs, Au-Steroid, or their combination for 24 h. After treatment, mTORC1 activity, as well as OCR and ECAR, were individually assessed (Figure 5A). OA chondrocytes with *Ddit4* knockdown exhibited stronger activation of mTORC1 during IL-1β stimulation compared to those in cells transfected with siControl. Consistent with the results shown in Figure 4, treatment with MSC-Au-Steroid in the siControl transfection group significantly reduced the phosphorylation levels of RAPTOR compared to those with MSC or Au-Steroid alone, and p70S6 phosphorylation was significantly inhibited only by MSC-Au-Steroid. However, in *Ddit4* knockdown cells, MSC-Au-Steroid failed to inhibit mTORC1 activation, unlike in the siControl-transfected cells (Figure 5B-C) and was unable to reduce the expression of MMP3 and MMP13, unlike in siControl cells (Figure S3D-E). In contrast, gain-of-function experiments demonstrated that DDIT4 overexpression alone was sufficient to suppress mTORC1 activity. Forced expression of DDIT4 markedly reduced IL-1β-induced phosphorylation of RAPTOR and p70S6, thereby recapitulating the inhibitory effect of MSC-Au-Steroid on mTORC1 signaling (Figure S3F-G).

Furthermore, in *Ddit4*-knockdown cells, MSC-Au-Steroid failed to reverse the changes in mitochondrial metabolism and glycolysis profiles, as indicated by OCR and ECAR (Figure 5D-E). Taken together, these findings suggest that the inhibition of mTOR signaling and regulation of MMP production by MSC-Au-Steroid in OA chondrocytes are dependent on DDIT4. This suggests that *Ddit4* was a crucial target in the process by which MSC-Au-Steroid suppressed inflammation-related metabolic signaling in OA chondrocytes.

### MSC-Au-Steroid regulates OA pathogenesis in mice

To investigate the therapeutic effects of MSC-Au-Steroid on OA *in vivo*, we established a monosodium iodoacetate (MIA)-induced OA mouse model. This model includes cartilage and bone pathological features similar to those of human OA, and the disease progression and severity can be controlled by adjusting the MIA dosage, making it useful for evaluating the efficacy of DMOADs 30-32. The absence of *in vivo* toxicity of MSC, Au-Steroid, and MSC-Au-Steroid was confirmed before treatment. A comprehensive safety evaluation involved assessing body weight changes, gross morphology, and histopathological features of major organs, including the colon, kidney, liver, spleen, lung, and lymph nodes. Serum biochemical analyses were performed to measure alanine aminotransferase, aspartate aminotransferase, total bilirubin, total protein, albumin, albumin-to-globulin ratio, blood urea nitrogen, and creatinine levels. Hematological profiling included red and white blood cell counts, hemoglobin, hematocrit, mean corpuscular volume, mean corpuscular hemoglobin, mean corpuscular hemoglobin concentration, platelet count, and differential leukocyte analysis for neutrophils, lymphocytes, monocytes, eosinophils, and basophils. Additionally, T-cell subset profiling was conducted in the major organs to assess systemic immune responses. No significant abnormalities were observed in any of these evaluations, indicating that MSC, Au-Steroid, and MSC-Au-Steroid did not induce detectable toxicity *in vivo* (Figure S4A-G).

**Figure 6:**
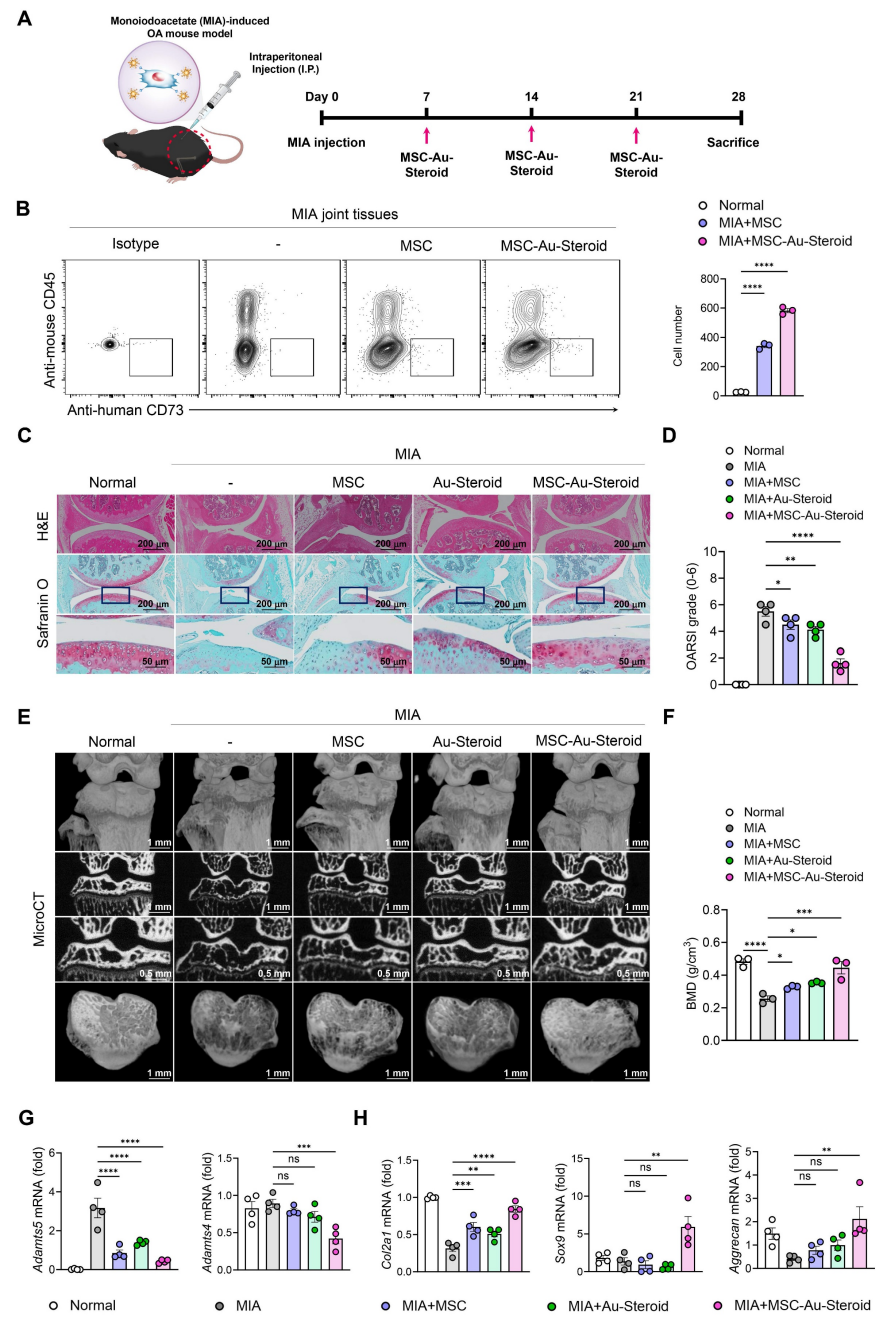
** MSC-Au Steroid ameliorates OA progression in an MIA-induced OA mouse model. (A-H)** C57BL/6J mice were injected intra-articularly with 0.75 mg MIA and then treated via intraperitoneal injection with MSC (1×10^6^ cells), Au-Steroid (3 mg/kg), or MSC (1×10^6^ cells)-Au-Steroid (3 mg/kg). All experiments were conducted on day 28. **(A)** Experimental design for MIA-induced OA mouse model. The mice were divided into five groups (*n* = 4 mice/group per experiment). **(B)** Flow cytometry analysis of digested knee joint tissues on day 28. Human MSCs were identified as mouse CD45⁻ human CD73⁺ cells. Representative FACS plots (left) and quantification of delivered MSCs in joint tissues (right). **(C)** Histological evaluation of knee joints 28 days after MIA induction: Safranin O staining of cartilage and H&E staining of synovitis (×200 magnification). **(D)** OARSI score in the indicated mice 28 days after MIA induction. **(E)** Representative micro-CT images of knee joints. **(F)** Micro-CT analysis results of bone mineral density (BMD). **(G)** Adamts (*Adamts4* and* Adamts5*) in the knee joints excised on day 28 were analyzed using qPCR. **(H)** Gene expression of cartilage biosynthesis markers (*Col2a1*, *Sox9,* and* Aggrecan)* in the knee joints excised on day 28 was analyzed using qPCR. Data are presented as the mean ± SEM. ****p < 0.0001, ***p < 0.001, **p < 0.01, and *p < 0.05.

Based on these findings, the treatments were subsequently administered to MIA-induced OA mice via intraperitoneal injection once a week for 3 weeks (Figure 6A). To confirm that the administered cells, including MSCs, were successfully delivered to the joint tissue, we analyzed digested knee joints on day 28 using flow cytometry. Human MSCs were identified as CD45⁻CD73⁺ cells, and the number of delivered cells was significantly higher in the MSC-Au-Steroid group compared to the MSC group (Figure 6B). The therapeutic efficacy of Au-Steroid, MSC, and MSC-Au-Steroid was evaluated based on the clinical symptoms associated with inflammatory responses. On day 28 after MIA induction, the Osteoarthritis Research Society International (OARSI) score increased to approximately 6 in the MIA group compared to that in the normal mouse group, whereas that in the MSC-Au-Steroid treatment group significantly decreased to 2 (Figure 6C-D). We also analyzed the osteophyte maturity, subchondral bone plate (SBP) thickness, and synovitis score, all of which increased with disease progression. Histopathological analysis showed that osteophyte formation, SBP thickness, and synovitis were significantly reduced in the MSC-Au-Steroid treatment group compared to those in the MIA-induced group, indicating that the cartilage structure damaged by MIA was well preserved by MSC-Au-Steroid (Figure S5A). Next, we analyzed the bone mineral density (BMD) and bone tissue morphology using micro-computed tomography (micro-CT) (Figure 6E). The MSC-Au-Steroid treatment group showed the highest BMD, similar to that of normal mice. Additionally, in the analysis of bone tissue morphometric indicators, bone volume (BV), bone volume to total volume ratio (BV/TV), trabecular thickness (Tb.Th), and trabecular number (Tb.N) were significantly increased in the MSC-Au-Steroid treatment group compared to that in the MSC or Au-Steroid single-treatment groups, whereas trabecular separation (Tb.Sp) was decreased (Figure 6F; Figure S5B). Thus, the MSC-Au-Steroid treatment group exhibited the most normal knee structure.

We also analyzed the mRNA and protein expression levels of key inflammatory and cartilage degradation factors related to OA in the knee joints. qPCR analysis showed that MSC-Au-Steroid significantly reduced inflammatory cytokines (*Tnfa*, *Il1b*, *Il6*, *Ifnγ*, and *Il17a*) and the chemokine* Cxcl12* in joints (Figure S5C), leading to a concomitant decrease in cartilage-degrading enzymes such as *Adamts4*, *Adamts5*, and MMPs (*Mmp3*, *Mmp9*, and *Mmp13*) (Figure 6G; Figure S5D). Notably, MSC-Au-Steroid reduced *Cxcl12* expression, suggesting a potential role in normalizing chemokine gradients in the joint microenvironment. Additionally, the MSC-Au-Steroid treatment group exhibited a marked increase in the expression of collagen type II alpha 1 chain (*Col2a1*), a major component of the cartilage matrix, as well as *Sox9* and *Aggrecan*, which are critical for maintaining cartilage structure (Figure 6H). Although the MSC and Au-Steroid single-treatment groups also showed some effects, they were less pronounced than those in the MSC-Au-Steroid treatment group.

To further validate the therapeutic efficacy of MSC-Au-Steroid in a progressive OA setting, we employed a surgically induced destabilization of the medial meniscus (DMM) model. Mice were monitored for 8 weeks, and treatments with MSC, Au-Steroid, or MSC-Au-Steroid were initiated from week 4 post-surgery (Figure S5F). Flow cytometry analysis confirmed that intraperitoneally administered human MSCs were able to survive in the joint tissues (Figure S5G). Histological evaluation demonstrated that MSC-Au-Steroid treatment significantly preserved proteoglycan content and reduced synovitis, resulting in lower OARSI scores compared with other groups (Figure S6H-I). Although overall bone mineral density was not completely restored, micro-CT analysis revealed partial recovery of subchondral bone structure in MSC-Au-Steroid-treated mice (Figure S6J-K). Furthermore, histopathological analysis showed that osteophyte formation, SBP thickness, and synovial inflammation were significantly reduced in the MSC-Au-Steroid group (Figure S6L). Consistently, micro-CT-based bone morphometric analysis demonstrated that BMD, BV, BV/TV, Tb.Th, and Tb.N were significantly increased, whereas Tb.Sp was decreased following MSC-Au-Steroid treatment, indicating preservation of subchondral bone architecture (Figure S6M). Together, these supplementary data confirm the therapeutic benefit of MSC-Au-Steroid in both chemically induced and surgically induced models of OA.

### MSC-Au-Steroid suppresses mTOR to preserve mitochondrial integrity and function in OA mice

**Figure 7:**
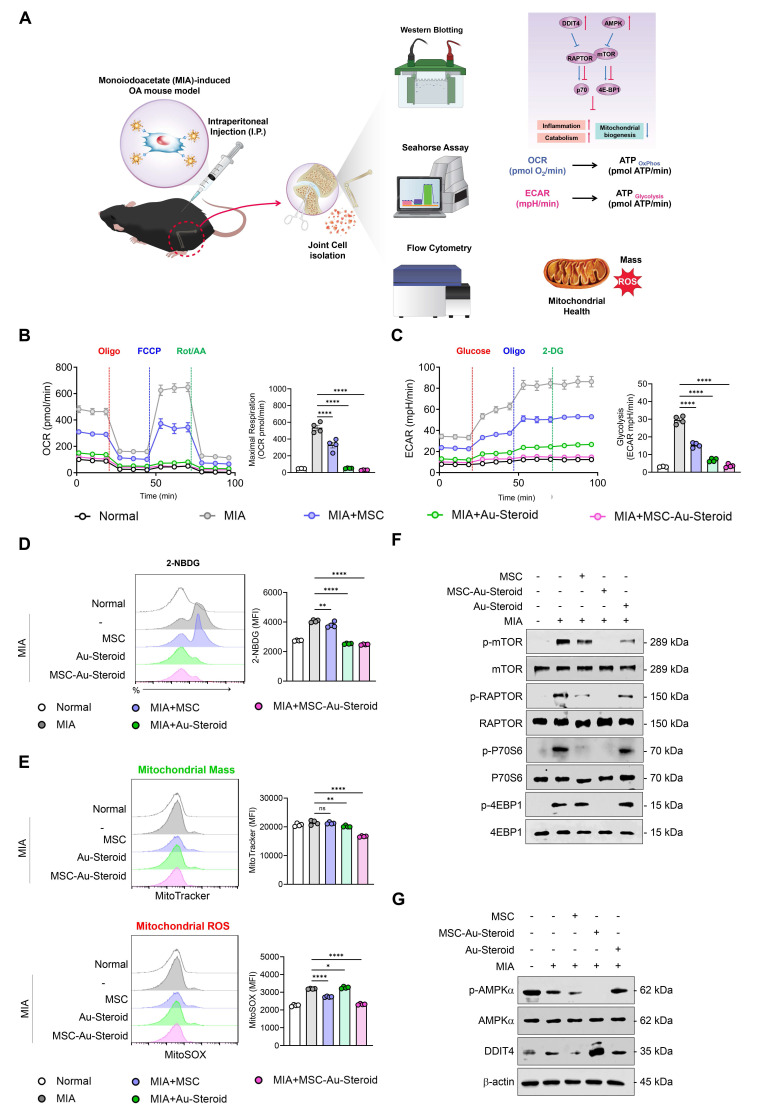
** MSC-Au-Steroid inhibits mTOR to maintain mitochondrial integrity and functionality in MIA-induced OA mice. (A-F)** C57BL/6J mice were intra-articularly injected with 0.75 mg MIA to induce OA and subsequently treated via intraperitoneal injection with MSCs (1 × 10⁶ cells), Au-Steroid (3 mg kg⁻¹), or MSC-Au-Steroid conjugates (1 × 10⁶ cells and 3 mg kg⁻¹). All analyses were performed 28 days after treatment. **(A)** Schematic illustration of the experimental design and workflow. **(B)** Real-time oxygen consumption rate (OCR) profiles of mouse knee joints following sequential administration of oligomycin, FCCP, and Rot/AA (left panels), with corresponding bar graphs showing maximal respiratory capacity (right panels). **(C)** Real-time extracellular acidification rate (ECAR) profiles of mouse knee joints in response to glucose, oligomycin, and 2-DG (left panels), with bar graphs indicating glycolytic activity (right panels). **(D)** Representative flow cytometry histograms and mean fluorescence intensity (MFI) analyses showing glucose uptake in MIA-induced mice treated with the indicated formulations. Glucose uptake was measured after incubation with 2-NBDG for 2 h followed by flow cytometric analysis. **(E)** Mitochondrial mass and mitochondrial ROS production in mouse joint cells analyzed by flow cytometry using MitoTracker Green (mitochondrial mass) and MitoSOX Red (mitochondrial ROS). Representative histograms (left) and MFI bar graphs (right) indicate relative mitochondrial activity. **(F)** Western blot analysis of mTOR and its downstream mTORC1 target proteins in mouse joint tissues. **(G)** Western blot analysis of AMPKα and DDIT4 expression in mouse joint tissues. Data are presented as mean ± SEM. ****p < 0.0001, ***p < 0.001, **p < 0.01, *p < 0.05.

Figures 2-4 suggest that MSC-Au-Steroid can reverse metabolic changes and protect the cartilage by normalizing dysfunctional mitochondria and inhibiting the mTOR pathway to ameliorate the inflammatory environment in OA chondrocytes. These results were reevaluated in the knee joints of OA mice, with the experimental workflow for intra-articular MSC-Au-Steroid administration and joint cell analysis shown in Figure 7A.

As observed in OA chondrocytes, knee joint cells isolated from mice with MIA-induced OA also showed significant increases in OCR (basal respiration, maximal respiration, and ATP production) and ECAR (glycolysis, glycolytic capacity, and glycolytic reserve) (Figure 7B-C; Figure S6A-B). These metabolic changes were significantly inhibited in all treatment groups of MSC, Au-Steroid, and MSC-Au-Steroid, which modulated mitochondrial respiration and the corresponding processes of inflammatory cells in the joints. Furthermore, glucose uptake (labeled with 2-NBDG) increased in the knee joints of MIA-induced OA mice but was reduced by MSC-Au-Steroid (Figure 7D). Furthermore, mitochondrial mass in knee joint cells was reduced in the MSC-Au-Steroid treatment group, which was confirmed by a decrease in MitoTracker fluorescence intensity (Figure 7E). Mitochondrial ROS levels were also significantly inhibited by MitoSOX (Figure 7E). Western blotting of mTOR and mTORC1 components showed that MSC-Au-Steroid treatment significantly downregulated the phosphorylation of mTOR, RAPTOR, p70S6K, and 4EBP1 in the knee joints of MIA-induced OA mice (Figure 7F; Figure S6C). Among AMPK and DDIT4, which were deregulated in OA mice, only DDIT4 was upregulated by the MSC-Au-Steroid treatment (Figure 7G; Figure S6D). These *in vivo* results are consistent with those already demonstrated *in vitro* and demonstrate that MSC-Au-Steroid reversed metabolic changes and exerted chondroprotective effects by normalizing dysfunctional mitochondria and inhibiting the mTOR pathway.

### MSC-Au-Steroid restores immune responses in OA

**Figure 8:**
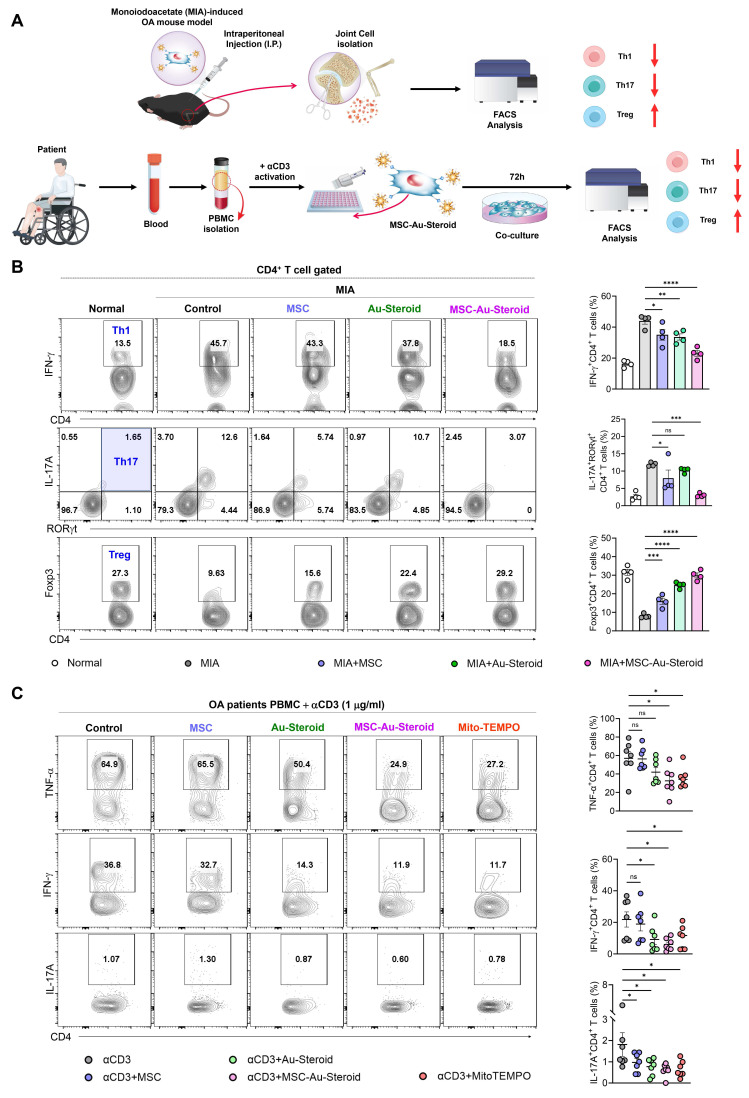
** Edu-MSC-Au-Steroid restores T cell immune responses in OA mice and patients. (A**) Schematic illustration of the experimental design depicting T-cell immune responses in knee joint cells from MIA-induced mice and PBMCs obtained from patients with OA. **(B)** Representative flow cytometry plots and quantification of CD4⁺IFN-γ⁺ (Th1), CD4⁺RORγt⁺IL-17A⁺ (Th17), and CD4⁺Foxp3⁺ (Treg) cells in the knee joint tissues (n = 4). **(C)** PBMCs isolated from OA patients were activated with anti-CD3 (1 μg mL⁻¹) for 72 h in the presence or absence of Mito-TEMPO (500 μg mL⁻¹), MSCs (1 × 10⁴ cells), Au-Steroid (500 ng mL⁻¹), or MSC-Au-Steroid conjugates (1 × 10⁴ cells and 500 ng mL⁻¹) (n = 7). Representative flow cytometry plots and bar graphs show the proportion of CD4⁺ T cells expressing TNF-α, IFN-γ, and IL-17A under the indicated conditions. Data are expressed as mean ± SEM. ****p < 0.0001, ***p < 0.001, **p < 0.01, *p < 0.05.

To confirm the restoration of the disrupted immune system in OA, we evaluated whether MSC-Au-Steroid regulated the innate and adaptive immune responses in knee joint cells from MIA-induced OA mice and in PBMCs from patients with OA (Figure 8A). Among inflammatory cells, macrophages and T cells increase in the peripheral blood, synovial fluid, and synovium of patients with OA and are associated with inflammation as well as joint dysfunction, including cartilage damage 33, 34. Flow cytometric analysis of knee joints from MIA-induced mice revealed a significant reduction in macrophages (F4/80^+^CD11b^+^) and M1 cells (F4/80^+^CD163^-^) in the MSC, Au-Steroid, and MSC-Au-Steroid treatment groups; however, no phenotypic shift to M2 macrophages (F4/80^+^CD163^+^) was observed (Figure S7A-B). Interestingly, despite the lack of a detectable M2 population as determined by flow cytometry, qPCR analysis revealed increased expression of several M2-associated genes, including Arg1 and IL-10, suggesting a partial activation of M2-related transcriptional programs (Figure S7C).

Next, we examined the rebalance of immune responses by analyzing changes in the generation of Th1 (CD4^+^IFN-γ^+^), Th17 (CD4^+^IL-17A^+^RORγT^+^), and Treg (CD4^+^Foxp3^+^) cells, which are key targets for immune recovery in OA. MSC-Au-Steroid most significantly suppressed Th1 and Th17 cells in the knee joint compared to MSC and Au-Steroid and successfully induced Tregs (Figure 8B). These results indicate that MSC-Au-Steroid prevented cartilage damage by inhibiting Th1/Th17 cells and effectively increasing the proportion and suppressive function of Treg cells, thereby promoting an immunoregulatory environment. Additionally, to confirm whether the increase in Th1 and Th17 cells in PBMCs from patients with OA resulted from ROS production, we activated T cell immune responses through CD3 activation and measured mitochondrial ROS levels using MitoSOX. The results showed that mitochondrial ROS levels were suppressed only in cells treated with MSC-Au-Steroid (Figure S8A). To support this, we compared the changes in Th1 (CD4^+^TNF-α^+^, CD4^+^IFN-γ^+^) and Th17 (CD4^+^IL-17A^+^) cells by treating activated T cells from both healthy individuals and patients with OA with the mitochondrial ROS inhibitor Mito-TEMPO, MSC, Au-Steroid, and MSC-Au-Steroid. In Th1 cells, TNF-α and IFN-γ were inhibited by MSC-Au-Steroid treatment, similar to the effects of Mito-TEMPO, whereas in Th17 cells, all treatments (MSC, Au-Steroid, and MSC-Au-Steroid) showed effects comparable to those of Mito-TEMPO (Figure 8C; Figure S8B-C). Importantly, OA PBMCs exhibited higher baseline frequencies of TNF-α⁺, IFN-γ⁺, and IL-17A⁺ CD4⁺ T cells compared with healthy PBMCs, and these inflammatory responses were effectively recovered by MSC-Au-Steroid (Figure S8D-E). To further assess whether MSC-Au-Steroid contributes to immune reprogramming through regulatory T cells, functional assays were performed using patient-derived Tregs. Co-culture of CD4⁺CD25⁻ effector T cells (Teff) with Tregs isolated from each treatment group demonstrated that MSC-Au-Steroid-derived Tregs exhibited the strongest suppressive capacity among all groups. Specifically, MSC-Au-Steroid treatment significantly reduced Teff proliferation, as determined by BrdU incorporation, and suppressed IFN-γ secretion in co-culture supernatants (Figure S8F-G). Together, these findings highlight the critical role of MSC-Au-Steroid in restoring immune regulatory functions in OA through inhibition of mitochondrial ROS production and reinforcement of Treg activity.

## Conclusion

OA is one of the most common types of arthritis. However, there is no fundamental cure for this disease to date. Despite advances in the understanding of OA pathology, clinical treatments remain focused on improving joint inflammation and pain rather than halting disease progression 5. In recent years, MSC have gained traction as DMOAD candidates, with a shift toward preventing massive cartilage destruction before it occurs and halting or delaying its progression through cartilage regeneration-promoting and anti-inflammatory effects 5, 10. However, MSC therapies face challenges in terms of addressing the root cause and demonstrating consistent efficacy 5, 10. As various signaling pathways and molecules may be involved in OA onset and progression, understanding and identifying potential biomarkers and therapeutic targets at different stages of OA progression is important 5. Over the past few decades, substantial evidence has suggested that metabolism plays a crucial role in maintaining energy homeostasis in articular chondrocytes and that metabolic abnormalities in chondrocytes can significantly affect a range of cellular functions, including signaling pathways, apoptosis, autophagy, and inflammation, ultimately leading to OA onset and progression 13, 22.

In this study, we conjugated MSC with gold nanoparticles-triamcinolone (Au-Steroid) to develop MSC-Au-Steroid, which exerts potent chondrolysis-inhibitory and regenerative effects and was proposed as a comprehensive solution to target the complex pathogenesis of OA by coordinating innate and adaptive immunity. Our results demonstrated that MSC-Au-Steroid exerted anti-inflammatory and chondrolysis-inhibitory effects on chondrocytes from patients with OA as well as therapeutic effects that induced immune recovery and cartilage regeneration. These effects were consistent with the results of our previous RA study, as MSC-Au-Steroid simultaneously targeted both innate and adaptive immune systems, which is a strength of MSC-Au-Steroid 12. In particular, they significantly decreased glucose uptake in the inflammatory environment of OA, suggesting that this treatment could maintain a healthy balance of cellular energy metabolism, increase chondrocyte survival, and reduce tissue damage.

The effectiveness of MSC-Au-Steroid was prominently demonstrated in the regulation of mitochondrial dysfunction and oxidative stress, which play critical roles in the progression of OA 35. OA chondrocytes undergo metabolic reprogramming, characterized by increased glucose uptake and corresponding processes, due to chronic inflammation. This leads to mitochondrial dysfunction and excessive ROS production, which in turn impairs cartilage regeneration 36, 37. MSC-Au-Steroid prevented the accumulation of damaged mitochondria by regulating mTOR and its subcomplex, the mTORC1 pathway, which is a central regulator of cellular metabolism. By upregulating DDIT4, MSC-Au-Steroid effectively maintained mitochondrial health and protected chondrocytes from oxidative damage by reducing ROS levels. To date, no reports have focused on the bioenergetic profiles of OA *in vivo* 35, suggesting that MSC-Au-Steroid could represent a novel therapeutic strategy to address multiple pathways of OA pathogenesis simultaneously by modulating metabolic imbalances.

Furthermore, the immunomodulatory effects of MSC-Au-Steroid were evident in the modulation of immune cell responses in OA. Macrophages and T-cells are the primary immune cells involved in cartilage damage and repair 38. In mouse models, joint tissues, and PBMCs from patients with OA, MSC-Au-Steroid immediately suppressed macrophages and Th1 and Th17 cells, creating an environment favorable for immune tolerance while inducing Treg cells to restore immune homeostasis. This immune-restorative function is crucial in preventing cartilage destruction and facilitating tissue repair and regeneration 38. Importantly, treatment with Au-Steroid or MSC alone did not achieve the same immune balance as MSC-Au-Steroid, further emphasizing the synergistic effect of combined treatment.

MSC-Au-Steroid also demonstrated superior effects in terms of cartilage regeneration by promoting anabolic processes in chondrocytes from patients with OA and in the joints of mouse models. MSC-Au-Steroid inhibited inflammatory cytokines such as TNF-α, IL-1β, and IL-6 induced by IL-1β, as well as matrix-degrading enzymes such as MMP3, MMP9, and MMP13, thus protecting chondrocytes from the inflammatory environment and contributing to maintaining the structural integrity of cartilage tissue. Furthermore, increased expression of cartilage regeneration-specific markers, such as Col2a, Sox9, and Aggrecan, indicated that MSC-Au-Steroid not only slowed disease progression but also promoted cartilage repair to a much greater extent than did Au-Steroid or MSC alone. This suggests that MSCs-Au-Steroid may offer a powerful advantage in OA by fundamentally suppressing inflammation while simultaneously contributing to cartilage regeneration.

Safety is critical for the development of new therapies and those that combine cells and nanoparticles present additional safety concerns 39. Consistent with previous results, MSC-Au-Steroid showed no toxicity, weight changes, or other adverse effects when administered repeatedly for 4 weeks in healthy mice 12. Our accumulated safety data indicate that MSC-Au-Steroid is a safe option for long-term treatment, providing important evidence for its potential clinical application.

In conclusion, our study demonstrates that MSC-Au-Steroid, a nano-cell hybrid therapeutic system, exerts robust disease-modifying effects in osteoarthritis by simultaneously targeting metabolic and immunological pathways. Specifically, MSC-Au-Steroid upregulated DDIT4 and inhibited mTORC1 signaling, preserving mitochondrial integrity, reducing ROS, and restoring chondrocyte metabolism. In parallel, it suppressed pathogenic Th1/Th17 responses while promoting Treg-mediated tolerance, thereby protecting cartilage and enhancing regeneration. These findings establish MSC-Au-Steroid as a promising DMOAD candidate that not only alleviates inflammation but also addresses the fundamental drivers of OA progression. Compared with conventional MSC or corticosteroid therapies, it provides a synergistic and integrative strategy with improved efficacy and safety. Collectively, this work offers a novel conceptual framework and a translationally relevant therapeutic platform for OA, underscoring the potential of nano-cell hybrid systems to overcome the limitations of current MSC-based treatments.

## Supplementary Material

Supplementary materials and methods, figures and tables.

## Abbreviations

OA: Osteoarthritis
NSAIDs: non-steroidal anti-inflammatory drugs
DMOADs: disease-modifying osteoarthritis drugs
MSC: Mesenchymal stem cell model educated to recruit to sites of inflammation
Steroid: conjugated the anti-inflammatory drug triamcinolone
Au: star-shaped gold nanoparticles
RA: Rheumatoid arthritis
ROS: reactive oxygen species
mTOR: mammalian target of rapamycin
mTORC1: mammalian target of rapamycin complex 1
ADAMTS5: A disintegrin and metalloproteinase with thrombospondin motifs 5
IL-1β: interleukin-1 beta
DDIT4: DNA-damage-inducible transcript 4
MMPs: Matrix metalloproteinase
TNF-α: tumor necrosis factor-α
FLSs: fibroblast-like synoviocytes
LPS: Lipopolysaccharides
PEG: Polyethylene Glycol
Abs: Anti-CD90 antibodies
Au-CD90-Steroid: Au-Steroid conjugated with CD90
FACS: Fluorescence-activated cell sorting
TEM: Transmission electron microscopy
STEM: scanning TEM
EDS: Energy-dispersive X-ray spectroscopy
S: sulfur, PEG
F: fluorine, TA
IL-6: interlukine-6
COL2A1: Collagen type II alpha 1 chain
SOX9: SRY-related HMG-box 9
Mmp1: Matrix metalloproteinase-1
Mmp3: Matrix metalloproteinase-3
Mmp9: Matrix metalloproteinase-9
Mmp13: Matrix metalloproteinase-13
ECAR: extracellular acidification rate
OCR: oxygen consumption rate
OXPHOS: oxidative phosphorylation
ATP: Adenosine triphosphate
2-NBDG: 2-(N-(7-Nitrobenz-2-oxa-1,3-diazol-4-yl)Amino)-2-Deoxyglucose
GLUT1: Glucose transporter 1
MitoTracker: mitochondrial membrane potential (Δψm)
MitoSOX: mitochondria-specific ROS
CMXROS: a Δψm-dependent mitochondrial dye
RAPTOR: Regulatory Associated Protein of mTOR
P70S6: Ribosomal protein S6 kinase beta-1
4EBP1: Eukaryotic Translation Initiation Factor 4E-Binding Protein 1
Ptpn1: Protein Tyrosine Phosphatase, Non-Receptor Type 1
Pten: Phosphatase and Tensin Homolog
Atg5: Autophagy Related 5
MIA: monosodium iodoacetate
OARSI: Osteoarthritis Research Society International
SBP: subchondral bone plate
BMD: bone mineral density
micro-CT: micro-computed tomography
BV: bone volume
BV/TV: bone volume to total volume ratio
Tb.Th: trabecular thickness
Tb.N: trabecular number
Tb.Sp: trabecular separation
Ifnγ: interferon-gamma
Il17a: interlukine-17A
AMPK: AMP-activated protein kinase
PBMCs: Peripheral Blood Mononuclear Cells
M1: M1 macrophage
M2: M2 macrophage
Th1: T helper type 1 cell
Th17: T helper type 17 cell
Teff: effector T cell
Treg: regulatory T cell
DMM: destabilization of the medial meniscus
mtDNA: mitochondrial DNA
