# Supplementary material for: Accelerated cartilage regeneration via chondrocyte metabolic reprogramming using nano-steroid-conjugated mesenchymal stem cells in osteoarthritis
Source: Theranostics. 2026 Jan 1;16(2):1023–44. doi: 10.7150/thno.120765 (PMC12675149; doi:10.7150/thno.120765)
Supplement: Supplementary file 1 — Supplementary materials and methods, figures and tables. [file thnov16p1023s1.pdf]

Supplementary Materials

Accelerated cartilage regeneration via chondrocyte metabolic reprogramming using nano-steroid-conjugated mesenchymal stem cells in osteoarthritis

Jong Yeong Lee, Jun Young Park, In Ah Kwon, Su Hyun Lim, Ki Bum Kim, Jun-Young Park, Youn Joo Kang, Sang-Hyun Kim, Dongwoo Khang, Jin Kyeong Choi

Corresponding author: Dongwoo Khang and Jin Kyeong Choi
E-mail: dkhang@gachon.ac.kr; jkchoi@jbnu.ac.kr

Files include:

Materials and Methods

Table S1 to S3

Figures S1 and S8

References

Materials and methods

Drug release profile

MSC-Au-Steroid complexes (1×10^6 cells) were individually prepared for each time point (0 h, 1 h, 6 h, and 12 h), and all samples were subsequently lyophilized for more than 24 hours to ensure stability. The dried complexes were dissolved in 100 μL of methanol and further diluted with an ACN:DW (50:50) mixture prior to analysis. Each sample was analyzed using a UPLC system (ACQUITY UPLC H-Class PLUS; Waters) equipped with a C18 column (1.7 μm, Waters) operated at 50 °C. The mobile phase conditions were programmed with a stepwise gradient, beginning with 100% DW, gradually increasing the ACN concentration over a defined period, and then returning to the initial condition for re-equilibration. The TA peak was consistently detected at 241 nm with a retention time (RT) of 28–32 min. All samples and mobile phases were filtered through a 0.2 μm PTFE-D filter prior to injection. Through this procedure, the drug release profile of MSC-Au-Steroid complexes was quantitatively evaluated across different time points.

Transmission Electron Microscope (Bio-TEM)

Human primary chondrocytes derived from OA patients were cultured for 24 h with or without IL-1β, MSCs, Au–Steroid, or MSC–Au–Steroid, following the method previously described. After incubation, the cells were fixed in a mixture of 2% glutaraldehyde (GA, EM grade) and 2% paraformaldehyde (FA, EM grade) prepared in 0.05 M sodium cacodylate buffer (pH 7.2) at 4 °C for 2 h. A secondary fixation step was performed using 1% osmium tetroxide for 1 h and 30 min. The fixed samples were rinsed three times with distilled water and incubated with 0.5% uranyl acetate at 4 °C for 30 min in the dark. After dehydration through graded ethanol, the specimens were treated with 100% propylene oxide and embedded in Embed 812 resin, followed by polymerization at 60 °C for 48 h. Ultrathin sections were prepared using an

ultramicrotome (UC7, Leica), mounted on copper grids, and visualized with a transmission electron microscope (H-7650, Hitachi).

DMM-induced mouse model of OA

Mice were under general anesthesia with 1.5–2% isoflurane (657801261, Hana pharm) prior to surgery. Hair around the knee was removed using an electric razor, and the area was disinfected three times alternately with 70% ethanol and povidone-iodine solution (A11A1720B0030, Dongin pharm). A heating pad (37°C) was used to prevent hypothermia during surgery. An approximately 5–7 mm anteromedial incision was made on the left knee to expose the knee joint. The medial meniscus and the medial meniscus-tibialis ligament were transected to induce medial meniscus destabilization. After suturing the surgical site, 20 mg/kg of amoxicillin (A8523, Sigma) was injected subcutaneously to prevent joint infection, and the induction period was continued for 10 weeks. MSC (1×10^6 cells), Au-Steroid (3 mg/kg), and conjugated MSC (1×10^6 cells)-Au-Steroid (3 mg/kg) were administered starting from the fourth week after induction, once every week. Each group of mice was divided into five groups, with seven mice per group. At week 10, mice were sacrificed, and joint tissues were collected and fixed in 4% formaldehyde for pathological examination and micro-CT analysis.

DDIT4 Overexpression Plasmid Construction and Transfection

Full-length human DDIT4 cDNA was cloned into the pcDNA3.1 vector under the CMV promoter (V79020, Thermo Fisher). The integrity and orientation of the insert were confirmed by Sanger sequencing. The resulting plasmid was transformed into E. coli DH5α (18265017, Thermo Fisher) and cultured under ampicillin selection. Plasmids were amplified and purified using an Endofree plasmid kit (12362, Qiagen). Primary human chondrocytes (passages 2–3) were seeded and cultured until they reached 70–80% confluence. For transfection, 2 μg of

plasmid DNA per well (6-well plate) was mixed with Lipofectamine 3000 reagent (L3000015, Thermo Fisher) at a 1:3 DNA-to-reagent ratio, following the manufacturer's protocol. Cells were incubated at 37°C in 5% CO_2 for 48 h.

T cell Co-culture Assay

$CD4^+CD25^-$ effector T cells (Teff) were negatively isolated from PBMCs of OA patients using magnetic bead separation (Miltenyi Biotec) according to the manufacturer's instructions. Regulatory T cells (Tregs; $CD4^+CD25^+$, 130-091-301, Miltenyi Biotec) were obtained from each experimental group (Untreated, MSC, Au-Steroid, MSC-Au-Steroid) by flow cytometry sorting. Teff and Tregs were co-cultured at a 1:1 ratio (1×10^5 cells each per well) in 96-well round-bottom plates. Control wells contained Teff cultured alone under identical conditions. Cells were stimulated with 1 μg mL^{-1} anti-CD3 (555336, BD Biosciences) for 72 h at 37°C in 5% CO_2. T cell proliferation was measured by BrdU incorporation assay (Roche) according to the manufacturer's protocol. Culture supernatants were collected at the end of the incubation period, and IFN-γ secretion was quantified using a human IFN-γ ELISA kit (eBioscience).

Table S1.

Quantification of TA and conjugation efficiency of AuS-TA prior to attachment to Edu-MSCs. The data include the mass of conjugated TA per Edu-MSCs measured by UV–vis (246 µg ± 30) and UPLC (241.13 µg ± 0.82), as well as the final TA:AuS ratio after Edu-MSCs conjugation (1 : 2.39 ± 0.42).

Edu-MSC-AuS-TA		
	UV-vis	UPLC
Percentage of conjugated TA per AuS (Before conjugated Edu-MSCs)	41.7% (± 7.20)	-
Mass of conjugated TA per Edu-MSCs (1 X 10^6)	246 µg (± 30.00)	241.13 µg (± 0.82)
Final TA: AuS ratio (After conjugated Edu-MSCs)	1: 2.39 (± 0.42)	-

Table S2. Clinical characteristics of enrolled patients with degenerative osteoarthritis

Kellgren-Lawrence (K-L) Grading System for Knee Osteoarthritis: The K-L grade assesses knee OA severity using X-ray imaging (1).

Grade 0 (No OA): Normal

Grade 1 (Doubtful OA): Possible osteophytes

Grade 2 (Mild OA): Definite osteophytes, possible joint space narrowing

Grade 3 (Moderate OA): Medium osteophytes, definite joint space narrowing

Grade 4 (Severe OA): Large osteophytes, severe joint space narrowing, osteosclerosis

Patient ID	Sex	Age	K-L grade
1	Female	67	IV
2	Female	63	IV
3	Female	79	IV
4	Male	67	III
5	Female	70	IV
6	Female	58	IV
7	Female	80	IV

Table S3. Forward and reverse primer sequence
Human

Gene	Forward primer (5'>3')	Reverse primer (5'>3')
Tnfa	GAGCTGAGAGATAACCAGCTGGTG	CAGATAGATGGGCTCATACCAGG G
Il1β	GCT GAT GGC CCT AAA CAG ATG AA	TGA AGC CCT TGC TGT AGT GGT G
Il6	CCC CTG ACC CAA CCA CAA AT	CAT TTG CCG AAG AGC CCT CA
MMP1	TGG ACC TGG AGG AAA TCT TG	AGT TCA TGA GCT GCA ACA CG
MMP3	TTC CTT GGA TTG GAG GTG AC	TGC CAG GAA AGG TTC TGA AG
MMP9	GCC ACT ACT GTG CCT TTG AGT C	CCC TCA GAG AAT CGC CAG TAC T
MMP13	CTATGGTCCAGGAGATGAA	AGAGTCTTGCCTGTATCCTC
Col2a1	TCTACCCCAATCCAGCAAAC	GTTGGGAGCCAGATTGTCAT
Sox9	ACTTGCACAACGCCGAG	CTGGTACTTGTAATCCGGGTG
Ddit4	GTT TGA CCG CTC CAC GAG CCT	GCA CAC AAG TGT TCA TCC TCA GG
Ptpn1	TGT CTG GCT GAT ACC TGC CTC T	ATC AGC CCC ATC CGA AAC TTC C
Pten	TGA GTT CCC TCA GCC GTT ACC T	GAG GTT TCC TCT GGT CCT GGT A
Atg5	GCA GAT GGA CAG TTG CAC ACA C	GAG GTG TTT CCA ACA TTG GCT CA
β-actin	AGAGCTACGAGCTGCCTGAC	AGCACTGTGTTGGCGTACAG

Mouse

Gene	Forward primer (5'>3')	Reverse primer (5'>3')
Tnfa	GGC AGG TCT ACT TTG GAG TCA TTG C	ACA TTC GAG GCT CCA GTG AAT TCG G
Il1β	CCA AAA GAT GAA GGG CTG CTT	TGC TGC TGC GAG ATT TGA AG
Il6	TCC AGT TGC CTT CTT GGG AC	GT CTG TTG GGA GTG GTA TC
Ifnγ	TCAAGTGGCATAGATGTGGAAGA A	TGGCTCTGCAGGATTTTCATG
Il17a	CTCAAAGCTCAGCGTGTCCAAAC A	TATCAGGGTCTTCATTGCGGTGG A
Adamts4	CAT CCG AAA CCC TGT CAA CTT G	GCC CAT CAT CTT CCA CAA TAG C
Adamts5	GCC ATT GTA ATA ACC CTG CAC C	TCA GTC CCA TCC GTA ACC TTT G
Col2a1	CAC ACT GGT AAG TGG GGC AAG ACC G	GGA TTG TGT TGT TTC AGG GTT CGG G
Sox9	ATG CTA TCT TCA AGG CGC TG	GAC GTC GAA GGT CTC AAT GT
Aggrecan	CTG TCT TTG TCA CCC ACA CAT	GAA GAC GAC ATC ACC ATC CAG
Mmp1	CCT TCC TTT GCT GTT GCT TC	AGC CCA AAT AAC TGC TGC AT
Mmp3	AAG TTC CTC GGG TTG GAG AT	TTT CAA TGG CAG AAT CCA CA
Mmp9	ACC ACA TCG AAC TTC GA	CGA CCA TAC AGA TAC TG
Mmp13	TGA TGG ACC TTC TGG TCT TCT GG	CAT CCA CAT GGT TGG GAA GTT CT
Cxcl12	GGA GGA TAG ATG TGC TCT GGA AC	AGT GAG GAT GGA GAC CGT GGT G
Arg1	AGG CGC TGT CAT CGA TTT CT	TGG AGT CCA GCA GAC TCA AT
Cd206	CTC TGT TCA GCT ATT GGA CGC	CGG AAT TTC TGG GAT TCA GCT TC
Il10	AGG CGC TGT CAT CGA TTT CT	ATG GCG TGA GGG AGA GCA TAG

β-actin	ACC CTA AGG CCA ACC GTG AA	ATG GCG TGA GGG AGA GCA TAG

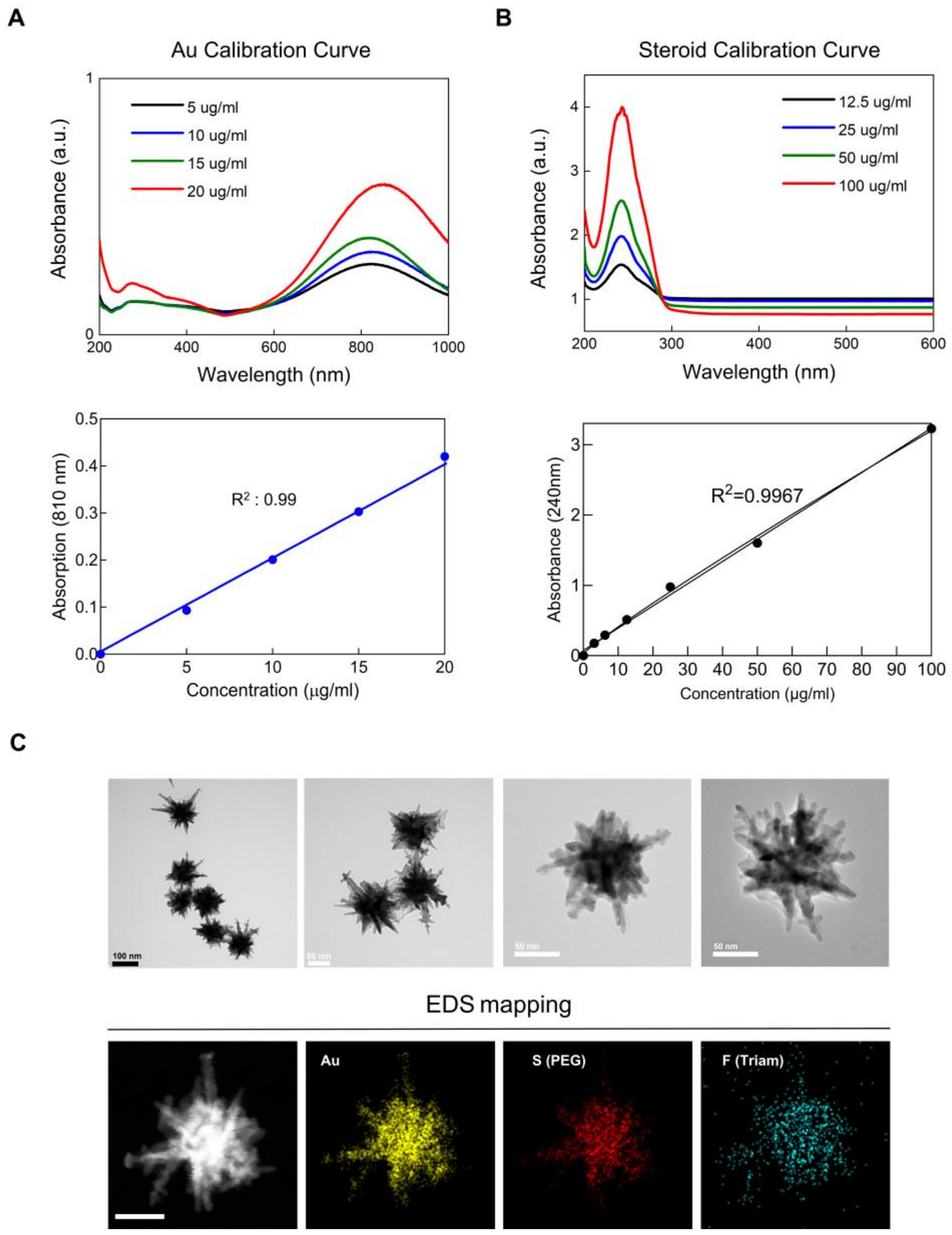
A
Au Calibration Curve
Absorbance (a.u.)
5 ug/ml
10 ug/ml
15 ug/ml
20 ug/ml
Wavelength (nm)
Absorption (810 nm)
R2 : 0.99
Concentration (μg/ml)
B
Steroid Calibration Curve
Absorbance (a.u.)
12.5 ug/ml
25 ug/ml
50 ug/ml
100 ug/ml
Wavelength (nm)
Absorbance (240nm)
R2=0.9967
Concentration (μg/ml)
C
100 nm
50 nm
50 nm
50 nm
EDS mapping
Au
S (PEG)
F (Triam)

D

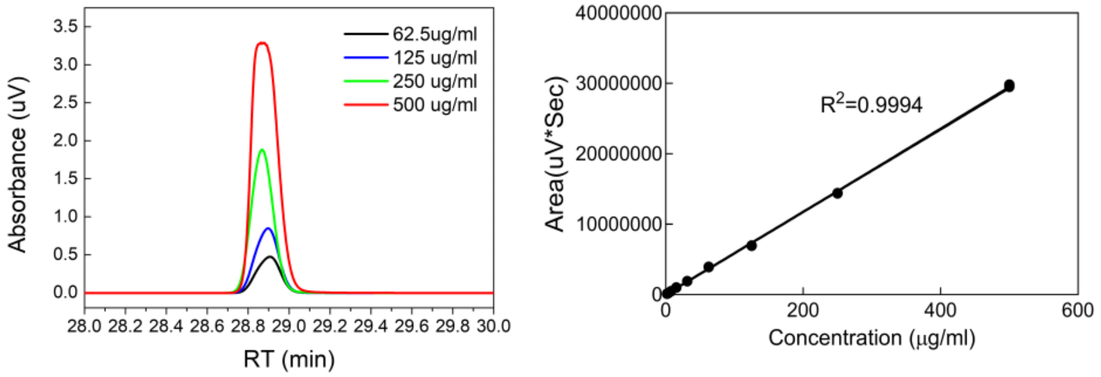

E

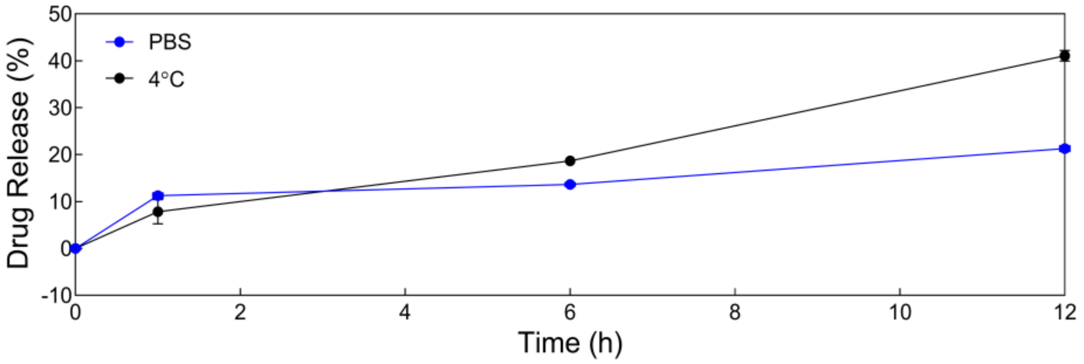

Figure S1. (A-B) Standard calibration curve for the quantification of Au and TA analyzed by UV–vis. **(C)** Transmission electron micrographs of Au-Steroid with energy-dispersive X-ray spectroscopy mapping (EDS mapping) showing the distribution of Au (yellow), S (red; from PEG), and F (blue; from Steroid). Scale bar (white) = 100 nm. **(D)** Standard calibration curve for the quantification TA analyzed by UPLC. **(E)** Time-dependent release profile of TA from MSC-Au-Steroid. Data are presented as mean ± SD (*n* = 3).

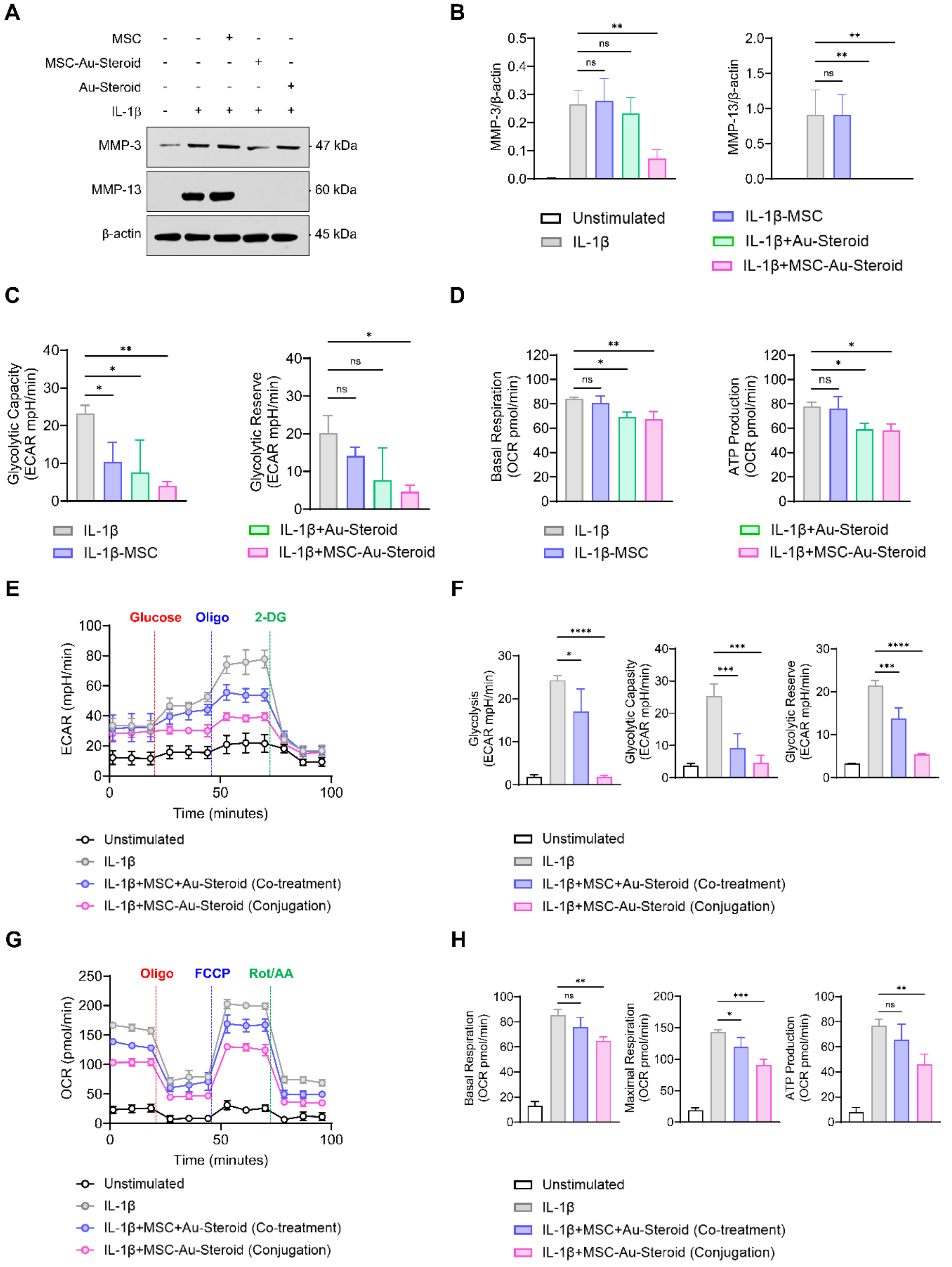

Figure S2. (A–H) OA chondrocytes were treated with IL-1β (10 ng mL^{-1}) for 24 h with or without MSCs (1 × 10^{4} cells), Au-Steroid (500 ng mL^{-1}), MSC plus Au-Steroid co-treatment,

or MSC-Au-Steroid conjugates ($n = 3$). **(A)** Western blot analysis of matrix metalloproteinases (MMP-1 and MMP-3). **(B)** Densitometric quantification of relative MMP protein levels. **(C)** Representative real-time ECAR traces of OA chondrocytes following sequential injections of glucose, oligomycin, and 2-deoxy-D-glucose (2-DG), with derived parameters for glycolytic capacity and glycolytic reserve. **(D)** Representative real-time OCR traces in response to oligomycin, FCCP, and Rot/AA, with bar graphs summarizing basal respiration and ATP-linked respiration. **(E)** Additional ECAR recordings for confirmation of glycolytic responses to glucose, oligomycin, and 2-DG. **(F)** Bar graphs summarizing glycolysis, glycolytic capacity, and glycolytic reserve. **(G)** Additional OCR recordings illustrating mitochondrial respiratory responses to oligomycin, FCCP, and Rot/AA. **(H)** Bar graphs summarizing basal and maximal respiratory capacity and ATP production. Data are presented as the mean ± SEM. ****$p < 0.0001$, ***$p < 0.001$, **$p < 0.01$, and *$p < 0.05$.

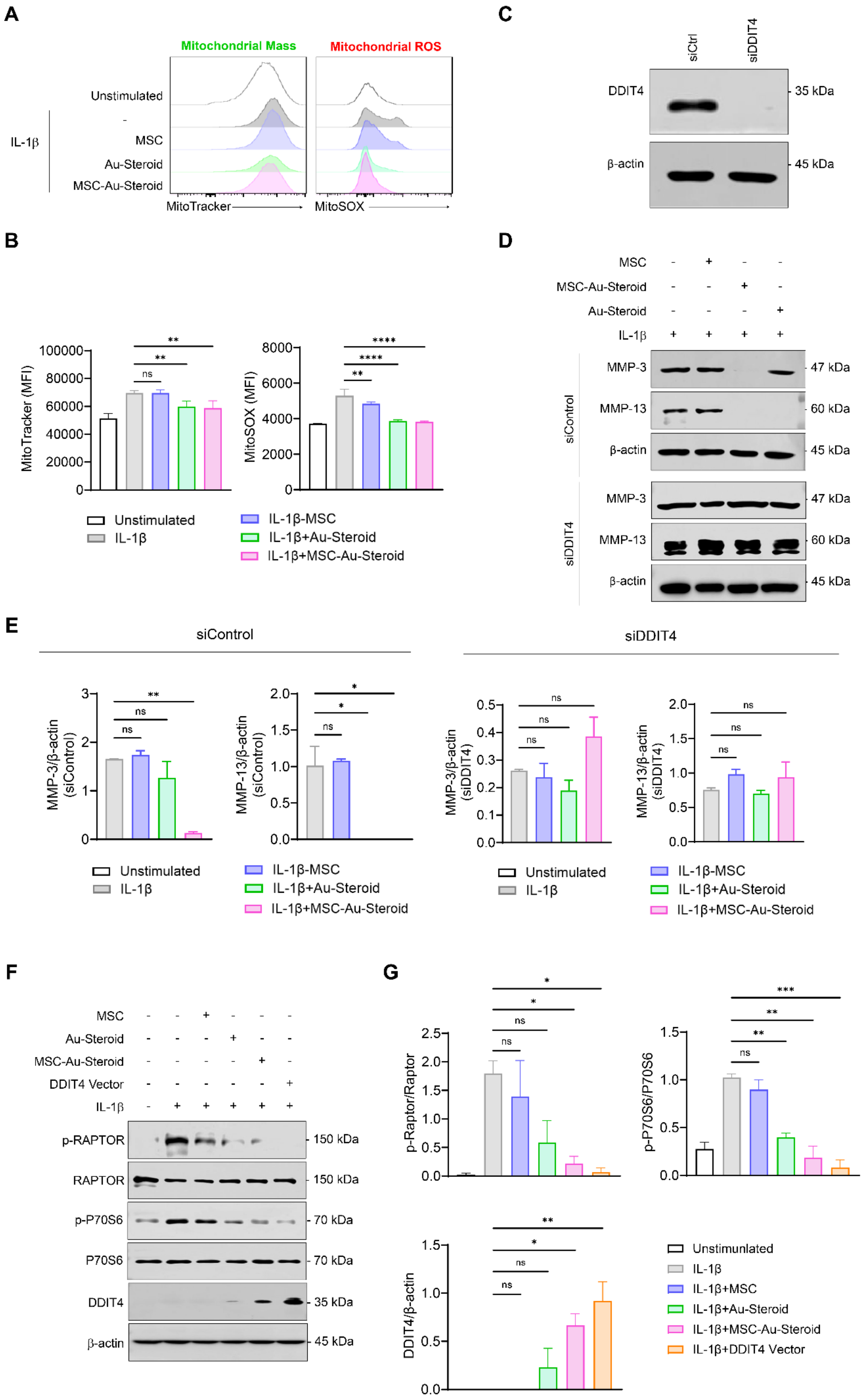
A
Mitochondrial Mass
Mitochondrial ROS
Unstimulated
IL-1β
MSC
Au-Steroid
MSC-Au-Steroid
MitoTracker
MitoSOX
B
MitoTracker (MFI)
MitoSOX (MFI)
Unstimulated
IL-1β
IL-1β-MSC
IL-1β+Au-Steroid
IL-1β+MSC-Au-Steroid
C
siCtrl
siDDIT4
DDIT4
β-actin
35 kDa
45 kDa
D
MSC
MSC-Au-Steroid
Au-Steroid
IL-1β
siControl
siDDIT4
MMP-3
MMP-13
β-actin
47 kDa
60 kDa
45 kDa
E
siControl
siDDIT4
MMP-3/β-actin (siControl)
MMP-13/β-actin (siControl)
MMP-3/β-actin (siDDIT4)
MMP-13/β-actin (siDDIT4)
F
MSC
Au-Steroid
MSC-Au-Steroid
DDIT4 Vector
IL-1β
p-RAPTOR
RAPTOR
p-P70S6
P70S6
DDIT4
β-actin
150 kDa
70 kDa
35 kDa
45 kDa
G
p-Raptor/Raptor
p-P70S6/P70S6
DDIT4/β-actin
Unstimunlated
IL-1β
IL-1β+MSC
IL-1β+Au-Steroid
IL-1β+MSC-Au-Steroid
IL-1β+DDIT4 Vector

Figure S3. (A-B) OA chondrocytes were stimulated with IL-1β (10 ng mL^{-1}) for 24 h in the presence or absence of MSCs (1×10^4 cells), Au–Steroid (500 ng mL^{-1}), or MSC–Au–Steroid conjugates (1×10^4 cells and 500 ng mL^{-1}) (n = 3). **(A)** Flow cytometric analysis of mitochondrial mass and mitochondrial ROS production in OA chondrocytes labeled with MitoTracker Green (mitochondrial content) and MitoSOX Red (mitochondrial ROS). **(B)** Mean fluorescence intensity (MFI) bar graphs showing mitochondrial mass (MitoTracker Green) and mitochondrial ROS levels (MitoSOX Red). **(C-E)** OA chondrocytes were transfected with control siRNA or DDIT4-specific siRNA for 48 h, followed by stimulation with IL-1β (10 ng mL^{-1}) in the presence or absence of MSCs (1×10^4 cells), Au–Steroid (500 ng mL^{-1}), or MSC–Au–Steroid conjugates (n = 3). **(C)** Western blot analysis of DDIT4 expression in chondrocytes transfected with control or DDIT4 siRNA. **(D)** Western blot analysis of MMP3 and MMP13 protein levels in control or DDIT4-silenced cells. **(E)** Quantification of the relative protein band intensities. **(F)** Western blot analysis of p-RAPTOR, RAPTOR, p-P70S6, P70S6, and DDIT4 expression in OA chondrocytes treated with MSCs, Au–Steroid, MSC–Au–Steroid, or DDIT4 vector under IL-1β stimulation. **(G)** Bar graphs showing the relative protein expression ratios of p-RAPTOR/RAPTOR, p-P70S6/P70S6, and DDIT4 normalized to β-actin.Data are presented as the mean ± SEM. ***$p < 0.001$, **$p < 0.01$, and *$p < 0.05$.

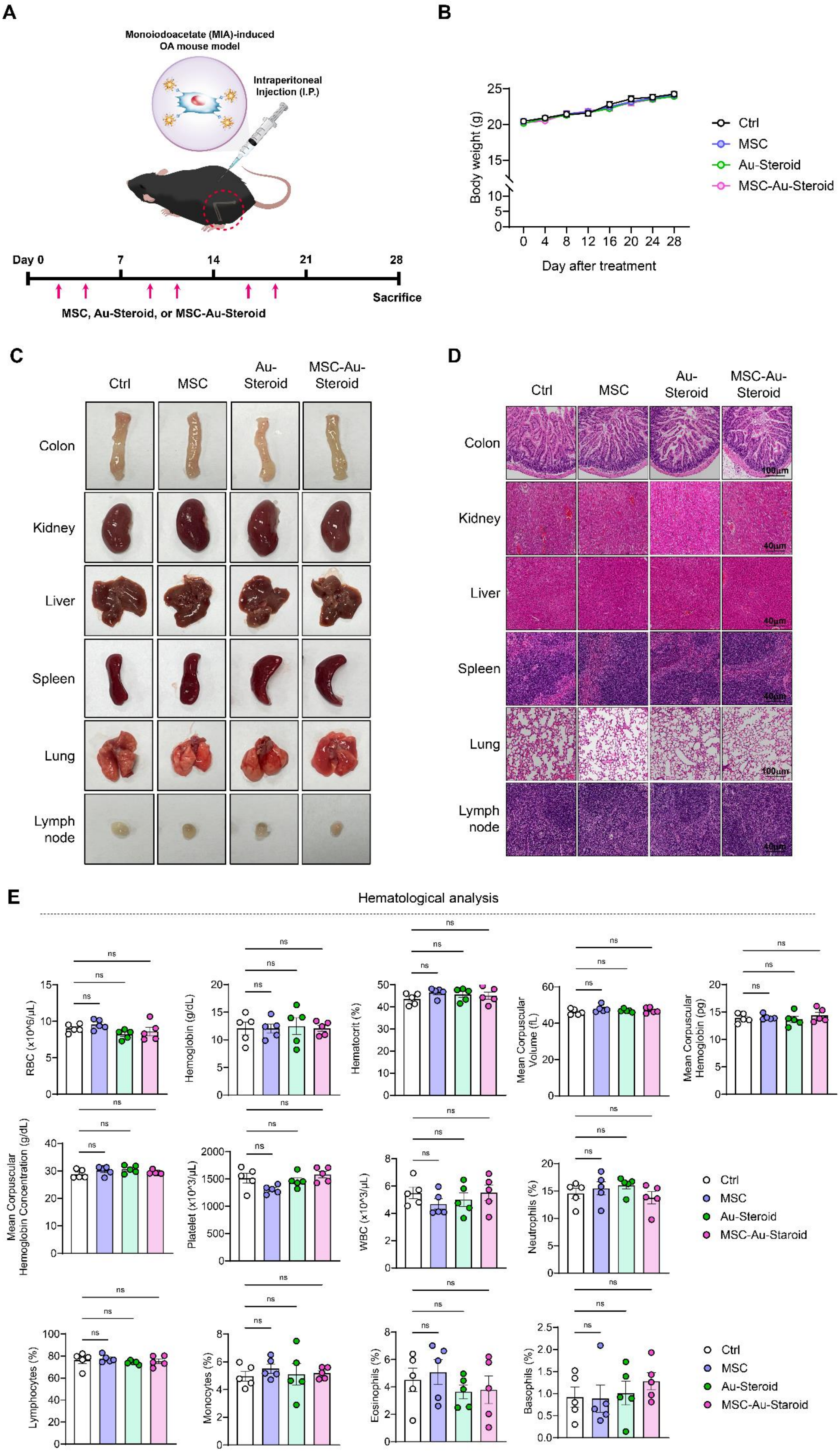

A
Monoiodoacetate (MIA)-induced
OA mouse model
Intraperitoneal
Injection (I.P.)
Day 0
7
14
21
28
Sacrifice
MSC, Au-Steroid, or MSC-Au-Steroid
B
Body weight (g)
Day after treatment
Ctrl
MSC
Au-Steroid
MSC-Au-Steroid
C
Ctrl
MSC
Au-Steroid
MSC-Au-Steroid
Colon
Kidney
Liver
Spleen
Lung
Lymph node
D
Ctrl
MSC
Au-Steroid
MSC-Au-Steroid
Colon
Kidney
Liver
Spleen
Lung
Lymph node
100μm
40μm
E
Hematological analysis
RBC (x10^6/μL)
Hemoglobin (g/dL)
Hematocrit (%)
Mean Corpuscular Volume (fL)
Mean Corpuscular Hemoglobin (pg)
Mean Corpuscular Hemoglobin Concentration (g/dL)
Platelet (x10^3/μL)
WBC (x10^3/μL)
Neutrophils (%)
Lymphocytes (%)
Monocytes (%)
Eosinophils (%)
Basophils (%)
ns
Ctrl
MSC
Au-Steroid
MSC-Au-Staroid

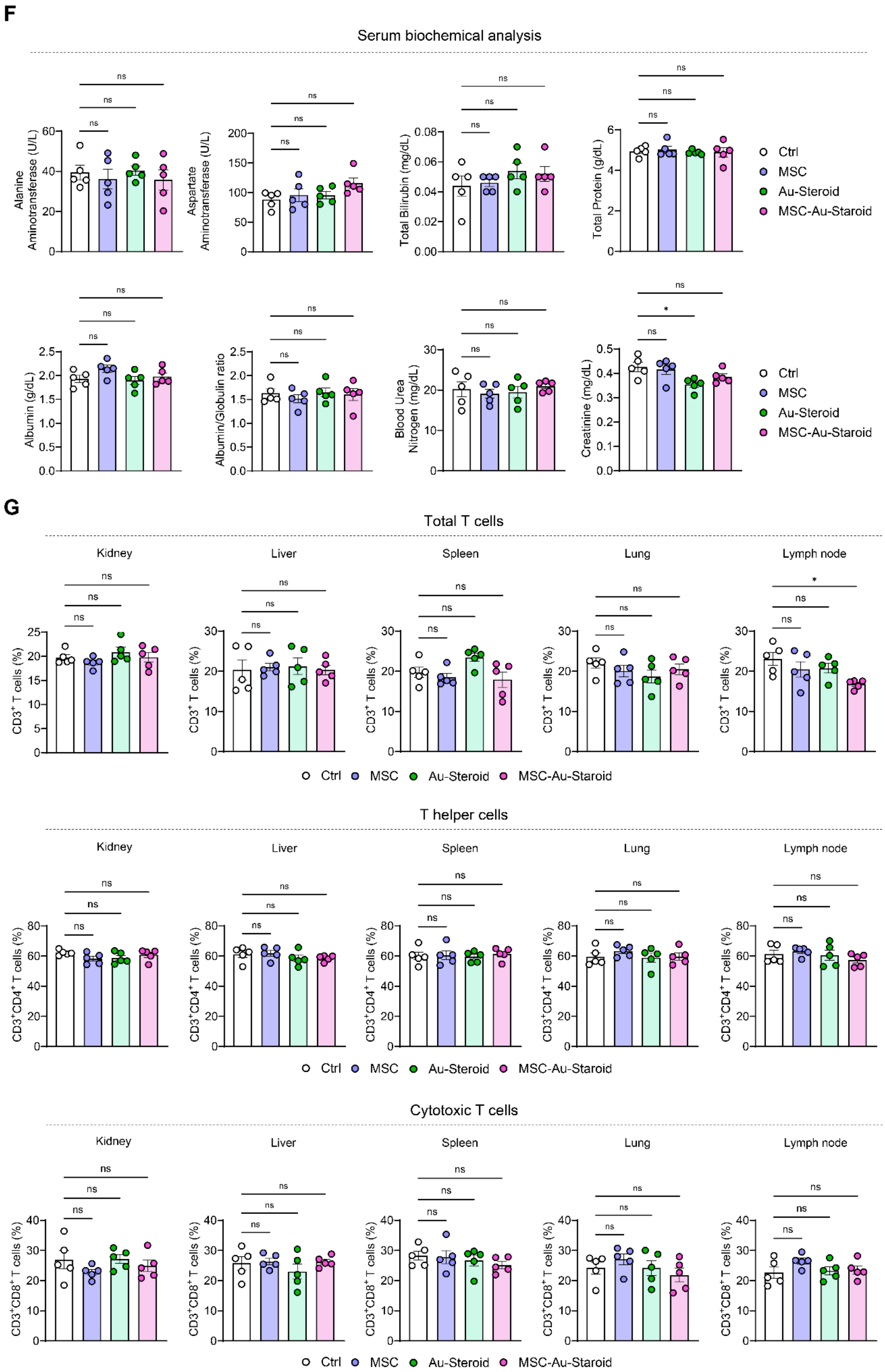
F
Serum biochemical analysis
Alanine Aminotransferase (U/L)
Aspartate Aminotransferase (U/L)
Total Bilirubin (mg/dL)
Total Protein (g/dL)
Albumin (g/dL)
Albumin/Globulin ratio
Blood Urea Nitrogen (mg/dL)
Creatinine (mg/dL)
ns
*
Ctrl
MSC
Au-Steroid
MSC-Au-Staroid
G
Total T cells
Kidney
Liver
Spleen
Lung
Lymph node
CD3+ T cells (%)
T helper cells
CD3+CD4+ T cells (%)
Cytotoxic T cells
CD3+CD8+ T cells (%)

Figure S4. (A) Schematic illustration of the repeated-dose toxicity study. C57BL/6 mice received intraperitoneal injections of MSCs (1×10^6 cells), Au–Steroid (3 mg kg^{-1}), or MSC-Au-Steroid conjugates (1×10^6 cells and 3 mg kg^{-1}) three times per week for 4 weeks to evaluate repeated-dose toxicity (n = 5). **(B)** Changes in body weight over the course of treatment. **(C)** Representative gross images of major organs collected from each treatment group. **(D)** Representative hematoxylin and eosin (H&E)–stained sections of the colon, kidney, liver, spleen, lung, and lymph node showing no histopathological abnormalities (×200 magnification). **(E)** Hematological analyses including red blood cell (RBC) count, hemoglobin (HGB), hematocrit (HCT), mean corpuscular volume (MCV), mean corpuscular hemoglobin (MCH), mean corpuscular hemoglobin concentration (MCHC), platelet (PLT) count, white blood cell (WBC) count, and differential leukocyte counts (neutrophils, lymphocytes, monocytes, eosinophils, and basophils). **(F)** Serum biochemical analyses including alanine aminotransferase (ALT), aspartate aminotransferase (AST), total bilirubin (T-bil), total protein (TP), albumin (Alb), albumin-to-globulin ratio (A/G), blood urea nitrogen (BUN), and creatinine (Crea). **(G)** Flow cytometric analysis of T-cell subsets ($CD3^+$, $CD4^+$, and $CD8^+$ T cells) in the kidney, liver, spleen, lung, and lymph node. No significant abnormalities were detected across groups, confirming the absence of systemic toxicity following repeated dosing.. Data are presented as the mean ± SEM.

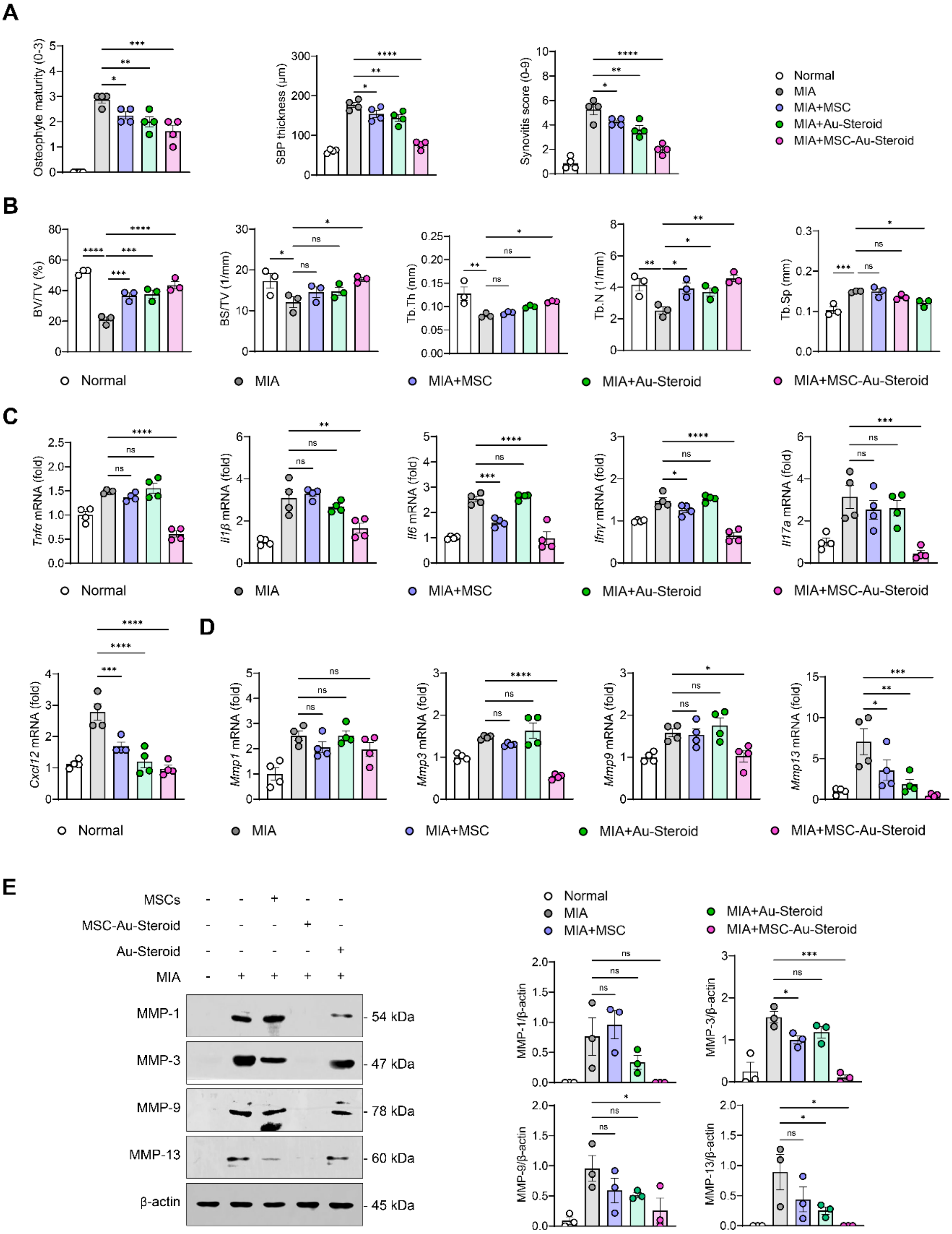

A
Osteophyte maturity (0-3)
SBP thickness (μm)
Synovitis score (0-9)
Normal
MIA
MIA+MSC
MIA+Au-Steroid
MIA+MSC-Au-Steroid
B
BV/TV (%)
BS/TV (1/mm)
Tb.Th (mm)
Tb.N (1/mm)
Tb.Sp (mm)
C
Tnfα mRNA (fold)
Il1β mRNA (fold)
Il6 mRNA (fold)
Ifnγ mRNA (fold)
Il17a mRNA (fold)
Cxcl12 mRNA (fold)
D
Mmp1 mRNA (fold)
Mmp3 mRNA (fold)
Mmp9 mRNA (fold)
Mmp13 mRNA (fold)
E
MSCs
MSC-Au-Steroid
Au-Steroid
MIA
MMP-1
MMP-3
MMP-9
MMP-13
β-actin
54 kDa
47 kDa
78 kDa
60 kDa
45 kDa
MMP-1/β-actin
MMP-3/β-actin
MMP-9/β-actin
MMP-13/β-actin

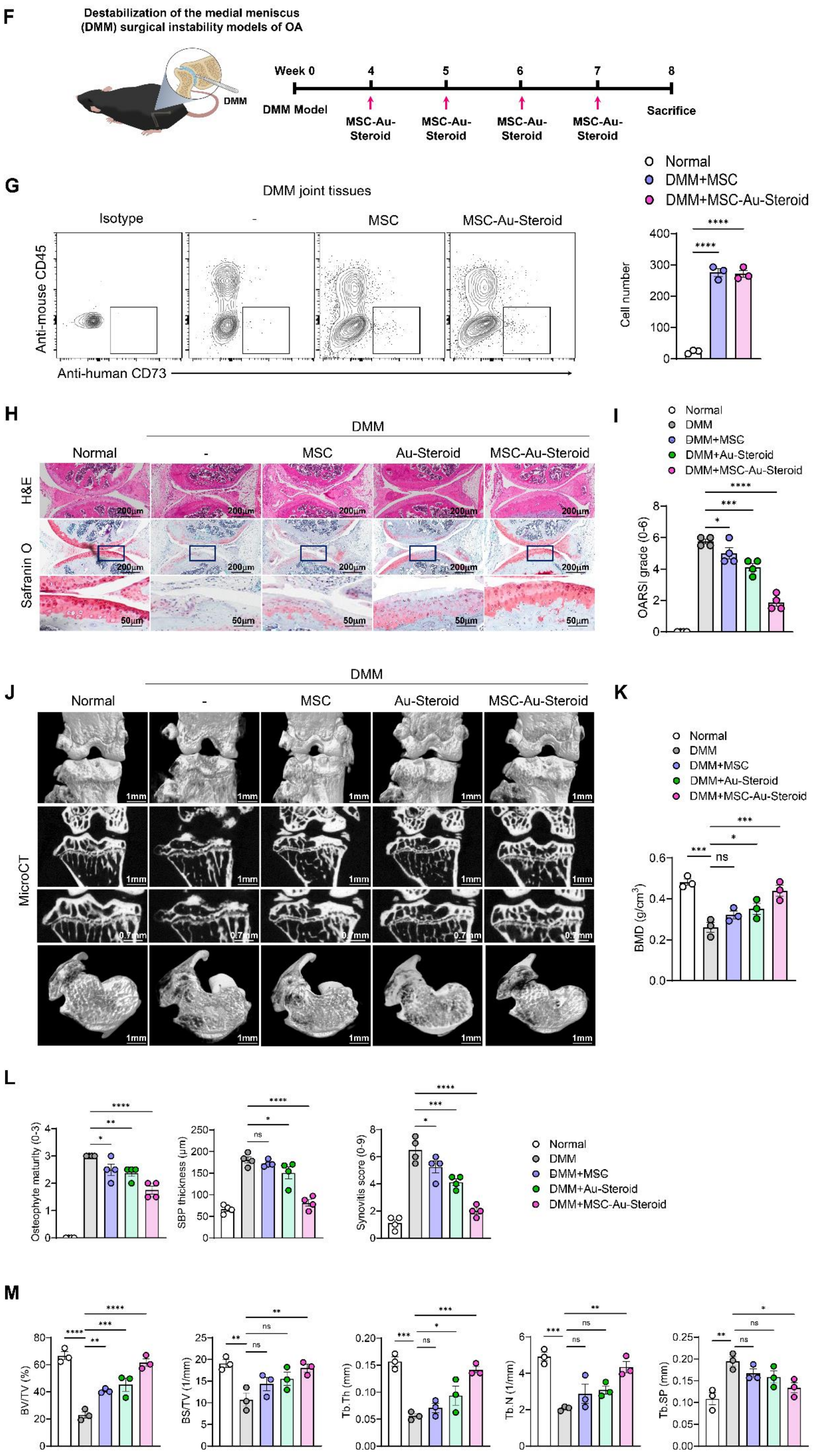
F
Destabilization of the medial meniscus
(DMM) surgical instability models of OA
DMM
Week 0
4
5
6
7
8
DMM Model
MSC-Au-Steroid
MSC-Au-Steroid
MSC-Au-Steroid
MSC-Au-Steroid
Sacrifice
G
DMM joint tissues
Isotype
-
MSC
MSC-Au-Steroid
Anti-mouse CD45
Anti-human CD73
Normal
DMM+MSC
DMM+MSC-Au-Steroid
Cell number
H
DMM
Normal
-
MSC
Au-Steroid
MSC-Au-Steroid
H&E
Safranin O
200μm
50μm
I
Normal
DMM
DMM+MSC
DMM+Au-Steroid
DMM+MSC-Au-Steroid
OARSI grade (0-6)
J
DMM
Normal
-
MSC
Au-Steroid
MSC-Au-Steroid
MicroCT
1mm
0.7mm
K
Normal
DMM
DMM+MSC
DMM+Au-Steroid
DMM+MSC-Au-Steroid
BMD (g/cm3)
L
Osteophyte maturity (0-3)
SBP thickness (μm)
Synovitis score (0-9)
Normal
DMM
DMM+MSC
DMM+Au-Steroid
DMM+MSC-Au-Steroid
M
BV/TV (%)
BS/TV (1/mm)
Tb.Th (mm)
Tb.N (1/mm)
Tb.SP (mm)

Figure S5. (A-E) C57BL/6J mice were injected intra-articularly with 0.75 mg MIA and then treated via intraperitoneal injection with MSC (1×10^6 cells), Au-Steroid (3 mg/kg), or MSC (1×10^6 cells)-Au-Steroid (3 mg/kg) (*n* = 4/group per experiment). **(A)** Osteophyte development, subchondral bone plate (SBP) thickness, and synovitis score in the indicated mice 28 days after MIA induction. **(B)** Micro-CT analysis results of bone volume fraction (BV/TV), bone surface to bone volume ratio (BS/TV), trabecular thickness (Tb.Th), trabecular number (Tb.N), and trabecular separation (Tb.Sp). **(C)** Gene expression of inflammatory cytokines (*Tnfα, Il1β, Il6, Ifnγ,* and *Il17a*), chemokine (*Cxcl12*), and **(D)** MMPs (*Mmp1, Mmp3, Mmp9,* and *Mmp13)* in the knee joints excised on day 28 was analyzed using qPCR. **(E)** Protein expression of MMPs was determined using western blotting (left panel) and bar graphs of relative intensities (right panel). **(F–M) (F)** Experimental design for the surgically induced DMM OA mouse model. C57BL/6J mice were subjected to DMM surgery and monitored for 8 weeks. Treatments with MSC (1×10^6 cells), Au-Steroid (3 mg/kg), or MSC (1×10^6 cells)-Au-Steroid (3 mg/kg) were administered intraperitoneally once a week starting at 4 weeks post-surgery, and analyses were performed at day 56 (*n* = 4/group per experiment). The mice were divided into five groups (*n* = 4 mice. **(G)** Flow cytometry analysis of digested knee joint tissues on day 56. Human MSCs were identified as mouse $CD45^-$ human $CD73^+$ cells. Representative FACS plots (left) and quantification of delivered MSCs in joint tissues (right). **(H-I)** Histological evaluation of knee joints after DMM induction. Safranin O staining was used to assess proteoglycan loss in articular cartilage, and H&E staining was used to evaluate synovitis (×200 magnification). Representative images are shown **(H)**, along with OARSI scores **(I)**. **(D)** OARSI score in the indicated mice 28 days after MIA induction. **(J-K)** Representative micro-CT images (J) and quantitative micro-CT analysis (K) of subchondral bone in DMM joints. Bone mineral density (BMD) was significantly improved in MSC-Au-Steroid–treated mice compared with other treatment groups. **(L)** Quantitative histological analysis of articular cartilage, including

Osteophyte development, subchondral bone plate (SBP) thickness, and synovitis score, performed on Safranin O-stained sections. **(M)** Micro-CT analysis results of bone volume fraction (BV/TV), bone surface to bone volume ratio (BS/TV), trabecular thickness (Tb.Th), trabecular number (Tb.N), and trabecular separation (Tb.Sp). Data are presented as the mean ± SEM. ****$p < 0.0001$, ***$p < 0.001$, **$p < 0.01$, and *$p < 0.05$

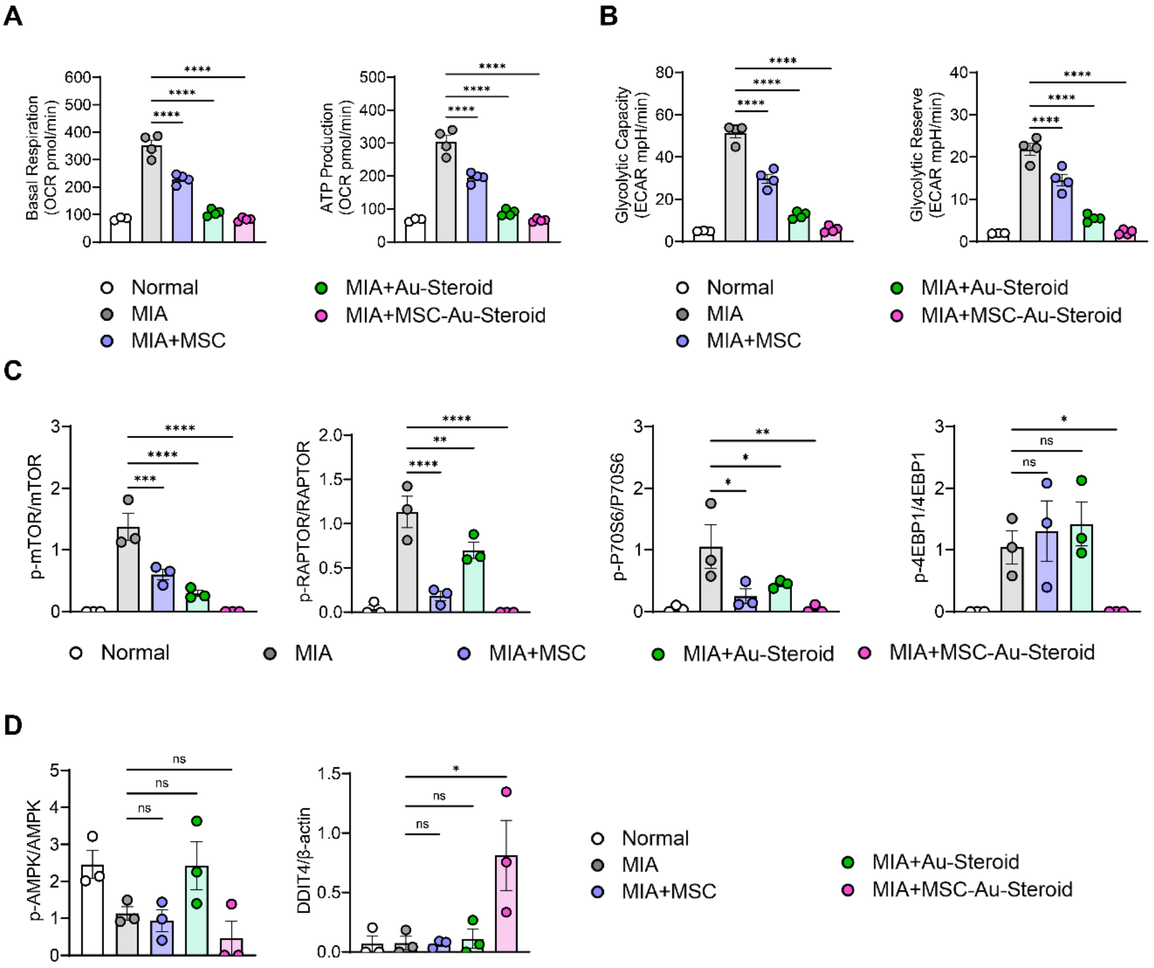

Figure S6. C57BL/6J mice were intra-articularly injected with 0.75 mg MIA to induce OA and subsequently treated via intraperitoneal injection with MSCs (1×10^6 cells), Au–Steroid (3 mg kg^{-1}), or MSC–Au–Steroid conjugates (1×10^6 cells and 3 mg kg^{-1}) (n = 4). **(A)** Real-time oxygen consumption rate (OCR) profiles of mouse knee joints in response to oligomycin, FCCP, and Rot/AA, with bar graphs showing basal respiration and ATP-linked respiration. **(B)** Real-time extracellular acidification rate (ECAR) profiles of mouse knee joints following sequential injections of glucose, oligomycin, and 2-DG, with corresponding bar graphs illustrating glycolytic capacity and glycolytic reserve. **(C)** Western blot analysis of mTOR and its downstream mTORC1 target proteins, with quantification of relative band intensities. **(D)** Western blot analysis of AMPKα and DDIT4 expression, with bar graphs representing relative protein intensities.

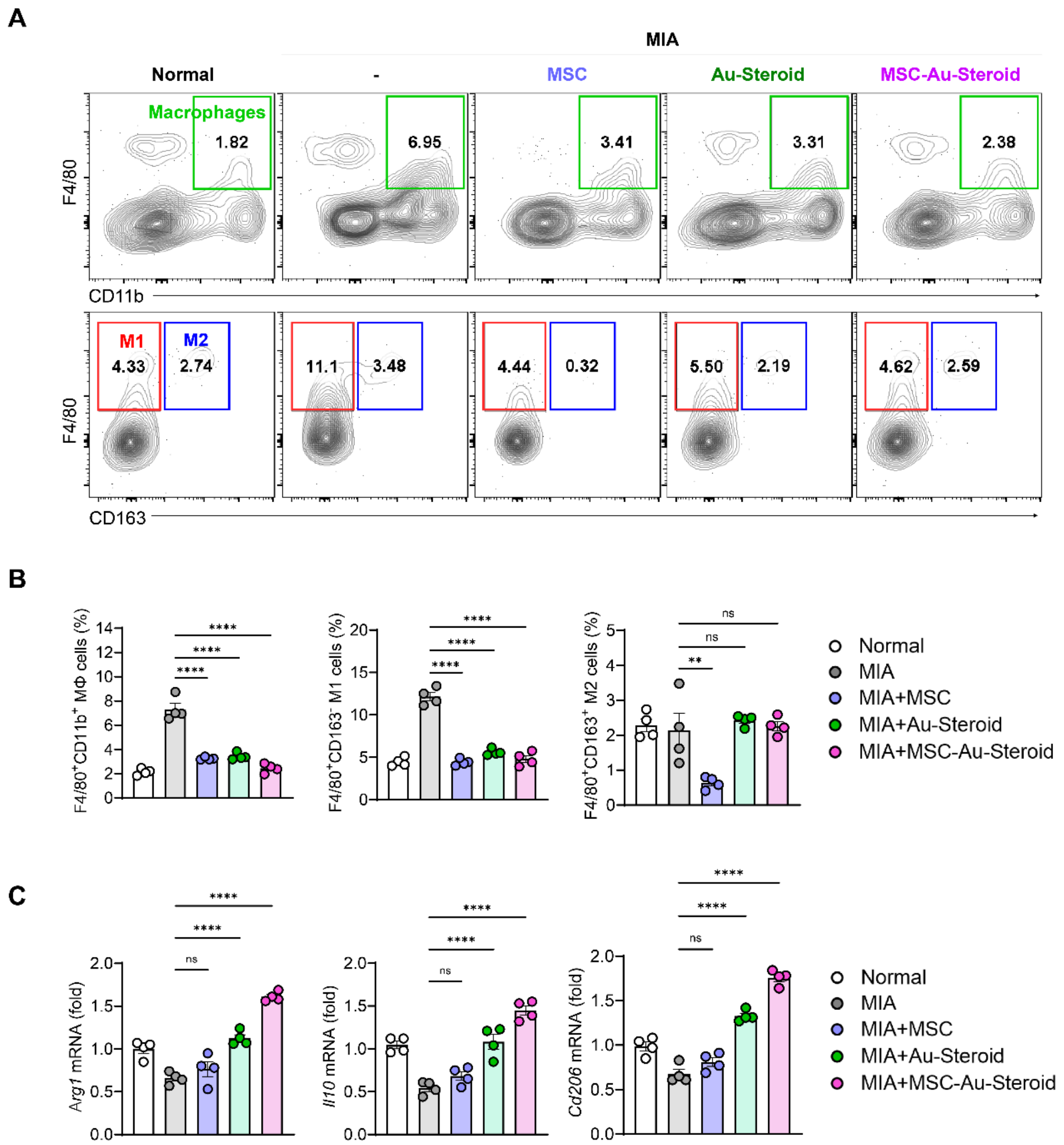

Figure S7. (A) Representative flow cytometry plots and **(B)** bar charts showing the percentages of F4/80^{+}CD11b^{+} (macrophages), F4/80^{+}CD163^{-} (M1 macrophages), and F4/80^{+}CD163^{+} (M2 macrophages) in knee joint cells. **(C)** Gene expression M2 macrophages marker (*Arg1*, *Il10*, and *Cd206)* in the knee joints excised on day 28 was analyzed using qPCR. Data are presented as the mean ± SEM. ****p < 0.0001, and **p < 0.01.

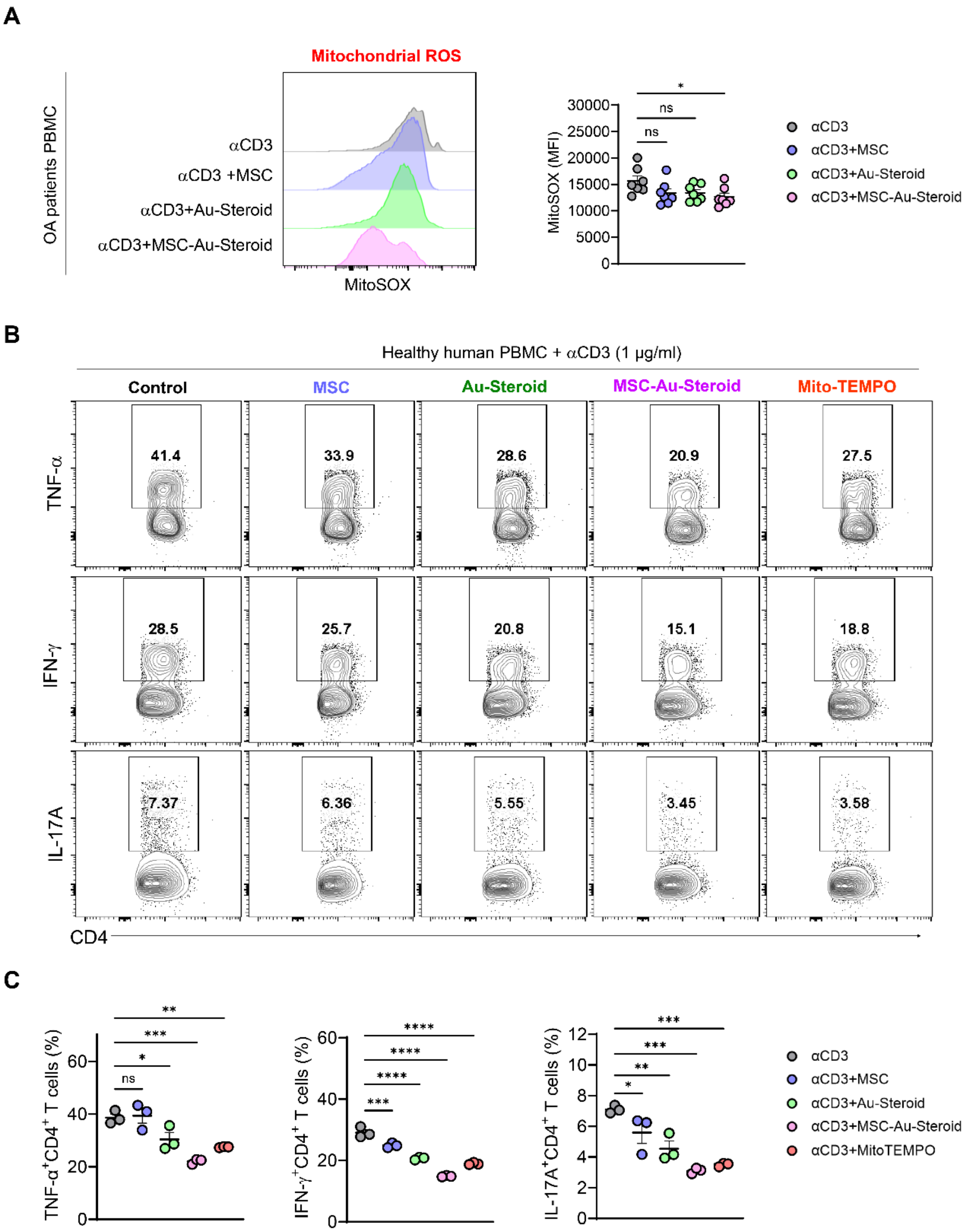
A
Mitochondrial ROS
OA patients PBMC
αCD3
αCD3 +MSC
αCD3+Au-Steroid
αCD3+MSC-Au-Steroid
MitoSOX
MitoSOX (MFI)
30000
25000
20000
15000
10000
5000
0
*
ns
ns
αCD3
αCD3+MSC
αCD3+Au-Steroid
αCD3+MSC-Au-Steroid
B
Healthy human PBMC + αCD3 (1 μg/ml)
Control
MSC
Au-Steroid
MSC-Au-Steroid
Mito-TEMPO
TNF-α
41.4
33.9
28.6
20.9
27.5
IFN-γ
28.5
25.7
20.8
15.1
18.8
IL-17A
7.37
6.36
5.55
3.45
3.58
CD4
C
TNF-α+CD4+ T cells (%)
60
40
20
0
**
***
*
ns
IFN-γ+CD4+ T cells (%)
60
40
20
0
****
****
****
***
IL-17A+CD4+ T cells (%)
12
10
8
6
4
2
0
***
***
**
*
αCD3
αCD3+MSC
αCD3+Au-Steroid
αCD3+MSC-Au-Steroid
αCD3+MitoTEMPO

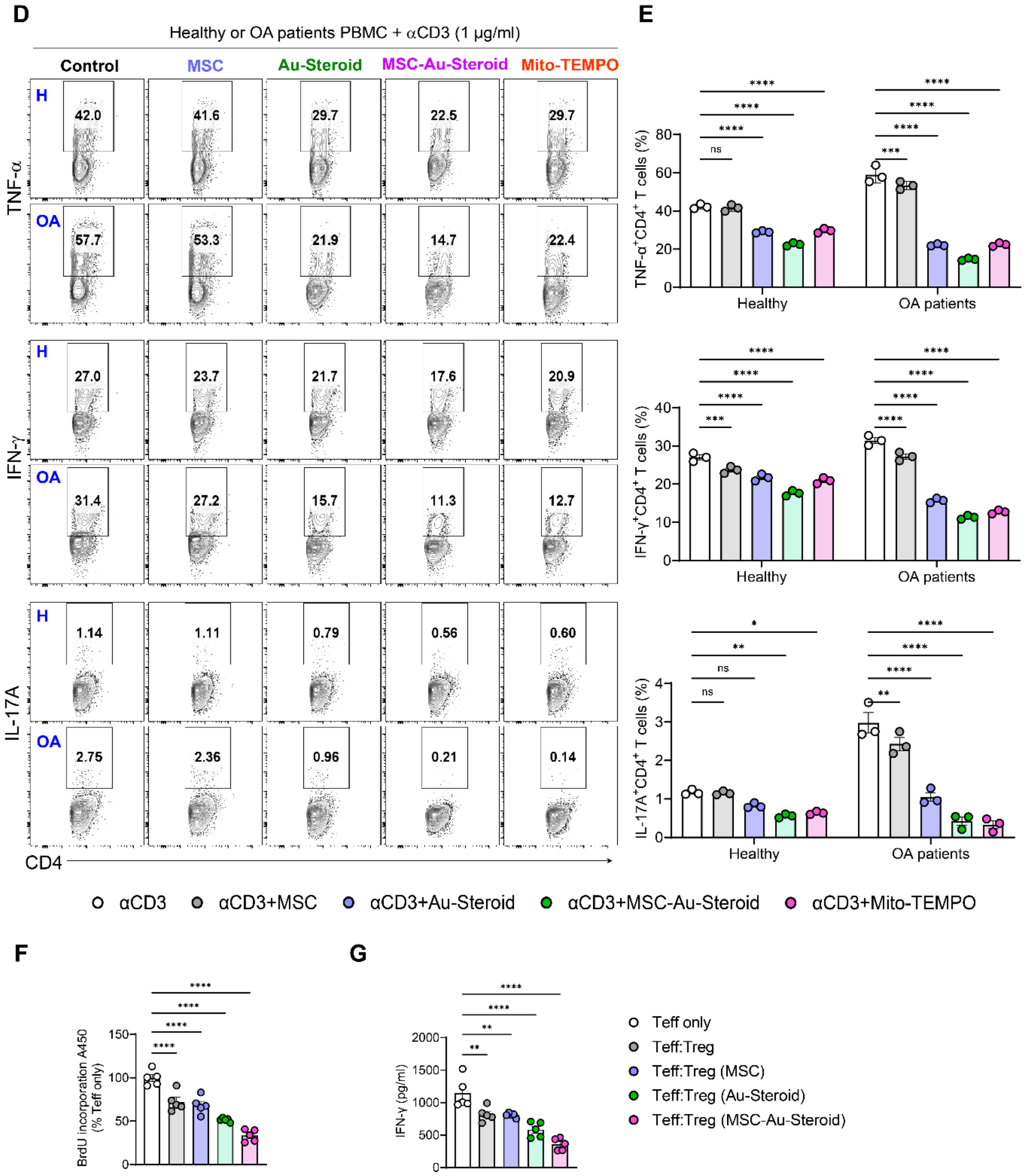

Figure S8. (A) Representative histograms (left) and mean fluorescence intensity (MFI) bar graphs (right) showing mitochondrial ROS levels in PBMCs from OA patients. T cells were stimulated with anti-CD3 (1 μg mL⁻¹) and treated with MSCs (1 × 10⁴ cells), Au–Steroid (500 ng mL⁻¹), or MSC–Au–Steroid conjugates (1 × 10⁴ cells and 500 ng mL⁻¹) for 72 h (n = 7). Cells were labeled with MitoSOX Red and analyzed by flow cytometry. **(B-C)** PBMCs isolated

from healthy donors were activated with anti-CD3 (1 μg mL^{-1}) in the presence or absence of Mito-TEMPO (500 μg mL^{-1}), MSCs (1×10^4 cells), Au–Steroid (500 ng mL^{-1}), or MSC–Au–Steroid conjugates (1×10^4 cells and 500 ng mL^{-1}) for 72 h (n = 3). Representative flow cytometry plots and corresponding bar graphs display the proportions of $CD4^+$ T cells expressing TNF-α, IFN-γ, and IL-17A. **(D-E)** PBMCs obtained from healthy donors (H, n = 3) or OA patients (OA, n = 3) were activated with anti-CD3 (1 μg mL^{-1}) and cultured for 72 h in the presence or absence of MSCs (1×10^4 cells), Au–Steroid (500 ng mL^{-1}), MSC–Au–Steroid (1×10^4 cells and 500 ng mL^{-1}), or Mito-TEMPO (500 μg mL^{-1}). Representative flow cytometry plots (D) and summarized bar graphs (E) show the frequency of $CD4^+$ T cells producing TNF-α, IFN-γ, and IL-17A. **(F-G)** Negatively magnetic-bead–purified $CD4^+CD25^-$ effector T cells (Teff) isolated from OA patients were cultured alone or co-cultured at a 1:1 ratio with flow cytometry–sorted $CD4^+CD25^+$ regulatory T cells (Tregs) derived from each treatment group (Untreated, MSC, Au–Steroid, MSC–Au–Steroid). Following 72 h stimulation with anti-CD3 (1 μg mL^{-1}), T cell proliferation was assessed by BrdU incorporation **(F)**, and IFN-γ production was quantified in culture supernatants by ELISA **(G)**. Data are presented as the mean ± SEM. ****$p < 0.0001$, ***$p < 0.001$, **$p < 0.01$, and *$p < 0.05$.

References

1. Sekiya I, Katano H, Guermazi A, Miura Y, Okanouchi N, Tomita M, et al. Association of AI-determined Kellgren-Lawrence grade with medial meniscus extrusion and cartilage thickness by AI-based 3D MRI analysis in early knee osteoarthritis. Sci Rep. 2023;13(1):20093.
